# Patients’ perspective about synchronous teleconsultation with a general practitioner: a mixed-method systematic literature review

**DOI:** 10.1186/s12875-025-02931-w

**Published:** 2025-08-20

**Authors:** Gabin F. Morillon, Marlène Guillon, Maude Laberge, Alphonse Sowanou, Thomas G. Poder

**Affiliations:** 1 Université de Montpellier, Montpellier Recherche en Economie, Avenue Raymond Dugrand, 34960 Cedex 2, Montpellier, France; 2 Fondation pour les Etudes et Recherches sur le Développement International (FERDI), 63 Boulevard François-Mitterrand, CS 50320, 63009 Clermont-Ferrand, France; 3 Faculty of Medicine, Université Laval, 1050 av de la Médecine, Québec, QC G1V 0A6 Canada; 4 Centre de recherche en santé durable, Vitam, Université Laval, Québec, QC Canada; 5 Faculty of Nursing, Université Laval, 1050 av de la Médecine, Québec, QC G1V 0A6 Canada; 6 Department of management, evaluation and health policy, School of public health, University of Montreal, 7101 Parc Avenue, Montreal, QC H3N 1X9 Canada; 7 Centre de recherche de l’IUSMM, CIUSSS de l’Est de l’Île de Montréal, Montréal, QC Canada

**Keywords:** Patient, Teleconsultation, Synchronous, General practitioner, Use, Experience, Satisfaction, Preference, Attitude

## Abstract

**Background:**

Synchronous teleconsultations using video or phone have become an increasingly popular method for delivering healthcare, especially in primary care. This modality enhances access to care, particularly for individuals in remote or underserved areas, and was especially significant during disruptions like the COVID-19 pandemic. Despite these benefits, patient perspectives on teleconsultations with general practitioners (GPs) remain underexplored. Factors such as consultation type, convenience, and technology influence patient satisfaction and acceptance, but concerns persist about the effectiveness of remote consultations for complex cases. This systematic review explores patients’ use, attitudes, experiences, satisfaction, and preferences regarding synchronous teleconsultations with GPs, aiming to identify factors influencing the choice of teleconsultation over face-to-face consultations.

**Methods:**

The review included 46 studies published until March 2023, sourced from PubMed, Web of Science, EBSCO, and Cochrane Library, following PRISMA guidelines. Quantitative, qualitative, and mixed-method studies were analyzed, representing diverse contexts.

**Results:**

Findings reveal that patient satisfaction with teleconsultation is influenced by consultation purpose, convenience, technological capabilities, and continuity of care. Video consultations are preferred over phone consultations, particularly for follow-ups and routine issues. Teleconsultation is seen as less effective for complex consultations requiring physical exams. Patient characteristics, including age, socioeconomic status, and technology familiarity, impact acceptance. Privacy concerns, data security, and diagnostic accuracy remain significant barriers. Continuity of care is better maintained when teleconsultation involves established patient-GP relationships.

**Conclusions:**

The review emphasizes the need for hybrid care models and policies aligned with patient preferences, focusing on accessibility, technology, and privacy safeguards. Future research should address barriers for vulnerable populations and equitable access.

**Supplementary Information:**

The online version contains supplementary material available at 10.1186/s12875-025-02931-w.

## Background

Telehealth could significantly enhance access to medical services, particularly for those facing barriers such as people living in rural areas. It can support continuous care for patients with chronic illnesses, offering an effective approach to ongoing health management [1]. By reducing regional disparities in care access and quality, telehealth also contributes to a more even distribution of medical expertise across different areas. It served as an essential alternative during the COVID-19 crisis too, ensuring patient care and monitoring that would be otherwise difficult to maintain [2]. Beyond merely providing remote patient care, telehealth adapts to the diverse needs of medical professionals, offering a variety of uses. Studies comparing telehealth practices across countries highlight its wide array of capabilities, such as optimizing patient flow in hospitals and improving the efficiency of appointment scheduling and document sharing. Furthermore, telehealth strengthens collaboration among healthcare providers (HCPs), leading to improved communication and coordination in patient care.

Among all practices surrounding telehealth, teleconsultation generally involves the use of information and communication technologies (ICTs) between a patient and a healthcare professional (HCP) in a remote healthcare setting [2–4]. By eliminating the notion of distance, teleconsultation helps to increase the supply of care, avoid patients giving up on care, particularly in areas where the supply of care does not meet all the population’s healthcare needs, and to facilitate access to care, meeting patients’ needs in the place where they live or nearby.

Today, teleconsultation is widely used, and decision-makers have already provided guidelines for its use [4–6]. Those guidelines emphasized many recommendations. To guarantee continuity and safety of care, it is advisable to alternate face-to-face and remote consultations, and for patients to live close to their doctor in case a face-to-face consultation is necessary. Although primary consultation is not a criterion excluding the use of teleconsultation, the latter is recommended when the relationship between patient and provider already exists. The World Health Organization (WHO) recommends that teleconsultation “*complement[s]*,* rather than replace[s]*,* the delivery of health services and in settings where patient safety*,* privacy*,* traceability*,* accountability and security can be monitored*” [1] A patient can use teleconsultation for any reason, although it is primarily considered for simple and routine reasons, such as prescription renewals. Teleconsultation must take place in a confidential setting, where the patient and HCP can talk freely. Finally, the patient must be able to carry out a teleconsultation, have sufficient knowledge or ability to call on the help of a relative. Consultation modality (i.e., phone or videoconference) is also an issue to consider. For instance, the French health authority recommends that teleconsultation should be performed by video [7]. However, the question arises as to whether these guidelines and the current use of teleconsultation are in line with patient preferences. In the context of patient-centered care, the consideration of preferences is essential to the establishment of healthcare policies.

Several literature reviews about telehealth were published and differed according to the type of telehealth, the modality of consultation, the medical specialty, and the health issue. Literature reviews by Agbali et al. (2021) [8] and Akesson et al. (2007) [9] covered several medical disciplines such as surgery, neurology, or pediatrics. Some literature reviews covered a wide range of modalities of consultation, asynchronous or synchronous (e.g., emailing, store and forwarding/internet platform [eConsult], phone or video) [9–12] while others focused specifically on synchronous modalities (i.e., phone or video) [8, 13–15]. Finally, a wide range of the literature focused on mental health (see [10] or [13]). However, to the best of our knowledge, no systematic review of the literature has focused solely on preferences regarding the consultation type between a patient and a general practitioner (GP), although the latter plays a pivotal role in many countries’ health systems. The aim of general practice is indeed to provide personal, comprehensive and continuous care for patients, without restrictions on disease, gender or age. Thus, consultations with GPs may concern significantly wider populations and multiple health issues (physical, mental, sexual or reproductive issues) with different specificities (e.g., already known, severe, or complex issues), as well as diagnosis or medical follow-up and may involve administrative tasks (e.g., prescription renewals, sickness certificates). The diversity of patients and conditions faced by GPs might pose specific challenges to the use of TC in this setting compared to specialized medicine. General practice is generally the patients’ first contact with the healthcare system, with GPs playing a key role in coordinating patient care with other physicians. Preferences for TC with a GP may shape preferences toward TC in other medical specialties; therefore, enhancing patient acceptance of TC in general practice could serve as a catalyst for broader adoption across the healthcare system.

Our systematic literature review aims to provide an overview of the literature on the determinants of patients’ use, satisfaction, attitude, experience, and preferences towards synchronous teleconsultation (i.e., by phone or videoconferencing) with a GP. More specifically, we aim to report on the determinants that favor the choice of a teleconsultation over a face-to-face consultation. This paper is organized as follows: Sect. 2 presents in detail the methodology we adopted from the selection of studies to reporting results, Sect. 3 presents our results by themes and sub-themes, Sect. 4 provides a summary of findings, policy implications, and an evaluation of the systematic literature review’s strengths and limitations, and Sect. 5 concludes.

## Methods

This mixed-method systematic review is part of two research projects conducted in parallel and in collaboration, and which aim to elicit preferences for synchronous teleconsultation with a GP, one in the French adult population and the other in the adult population of Quebec, Canada. To avoid the risk of bias from relying exclusively on quantitative studies, we also included qualitative and mixed-methods research, acknowledging their valuable contributions. Qualitative approaches are particularly effective in capturing individuals’ experiences and how they make sense of their realities within specific contexts. These insights complement quantitative findings by providing a deeper and context-sensitive interpretation of patient behaviors.

Two systematic reviews were conducted similarly on the same basis. The other systematic review focused on GPs’ perspective about synchronous teleconsultation (PROSPERO 2024 CRD42024505744). All the review process was performed considering both patients and GPs. We split both results at the end of the review process.

The Preferred Reporting Items for Systematic reviews and Meta-Analyses (PRISMA) statement was followed [16]. The protocol for the systematic review was registered on PROSPERO (2024 CRD42024467942). No amendment to the protocol was made. See Additional file 1.

The research team in charge of the systematic review was made up of specialists from several disciplines: health economics including health preference assessment and health technology assessment, health policy, primary care and family medicine. None of the team members had professional experience with teleconsultation, nor did they have any major positive or negative a priori.

This study was conducted with funding from the Canadian Institutes of Health Research, Project PJT 186,075, and from the Labex Entreprendre, University of Montpellier, France.

### Search strategy

The search strategy was performed on four databases (PubMed, Web of Science, EBSCO, and Cochrane Library) until March 23, 2023. The full search strategy is available in Additional file 2. Relevant keywords and associated truncations used in the searches were linked to the following concepts: teleconsultation, remote consultation, tele-medicine; general practitioner, family practitioner, general practice; and experience, satisfaction, use, preference, or attitude. The Boolean operators “AND” and “OR” were used to extend or refine search parameters, quotations marks (“”) to obtain results with exact expressions, and parentheses to group search terms. One member (GM) of the research team revised and integrated comments through an iterative process to develop a final version of the search strategy that was approved by all members.

### Selection criteria

We included studies that focused on the determinants of teleconsultation use, intention to use, attitude, and preferences, whether by phone or videoconference. The eligibility criteria for inclusion in our systematic review encompassed qualitative, quantitative, and mixed method studies, with no predefined exclusion criteria regarding study design. However, quantitative studies that presented only descriptive results without allowing for an examination of determinants’ effects on outcomes were excluded.

Exclusion criteria were applied to ensure the focus on studies presenting original data in detail, leading to the exclusion of reviews lacking new primary data, non-peer-reviewed studies, protocols, opinions, guidelines, editorials, letters, position papers, conference abstracts, correspondences, and comments. Teleconsultation was the central focus of our review. We specifically considered synchronous teleconsultation (via video or phone), excluding asynchronous methods like emailing or store-and-forwarding. Consultations with a treatment program nature, such as psychological follow-up, were also excluded. Additionally, studies on tele-expertise/e-consult, remote medical monitoring, and teleassistance were excluded. Our scope extended only to general/family medicine, excluding contexts like sports medicine or occupational medicine.

This systematic review included adult patients, along with their informal caregivers if part of the study, engaging in consultations with general practitioners, family physicians, community health physicians, and/or primary care physicians. We refer to the term “general practitioners” (GPs) in this study. These HCPs were selected as they represent common roles serving as the first point of contact for patient care, offering general care not confined to a specific medical field. The terminology varied across countries, using “general practitioner” in France, “family physician” (FP) in Canada, and “primary care physician” (PCP) in the United States. The exclusion of internists, trainees, and specialists was determined to maintain focus on GP.

No specific criteria were applied concerning the country of origin, encompassing both low- and middle-income countries as well as high-income countries. Additionally, there were no restrictions based on the date of the studies, nor on languages (non-English studies were transcribed using translation tools or with the help of native speakers).

The exclusion criteria used for abstract and full-text screening are outlined in Table 1.

**Table 1:** Exclusion criteria used for abstracts and full texts screening

1	Reviews without new primary data, protocols, not peer-reviewed, opinions, guidelines, editorial, letters, position papers, conference abstracts, case study [only one/two individuals], correspondence, and comments.
2	The study is not (exclusively) about synchronous (i.e., video and phone) teleconsultation. *Unsure that the subject of the study is synchronous teleconsultation. If telemedicine is mentioned but there is no certainty that it is teleconsultation*,* or if synchronous teleconsultation is mentioned but the results do not make it possible to differentiate it from other practices*,* then the article is excluded on this ground.*
3	Studies not investigating experience/use/satisfaction/attitudes. *The study does not cover the outcomes selected for the review.*
4	Studies do not investigate the barriers and benefits of teleconsultation from patients’ point of view. *The study does not allow to judge the direction of the relationship between the determinants and the outcomes selected. Quantitative descriptive studies that report only univariate results are excluded based on this criterion. The point here is to be able to ascertain the importance of teleconsultation characteristics and/or their effects on outcomes.*
5	The provider is not clearly defined, or results are not GP/FP/PCP specific. *The study covers a range of healthcare professionals*,* including those selected for the review or a broader set*,* but does not allow us to differentiate results between the different types of practitioners. Trainee practitioners are excluded.*

### Outcomes retained

We retained five outcomes: satisfaction, use and intention to use, attitude, experience, and preferences. These outcomes assess the patient’s care pathway, focusing on their needs and wishes, central to patient-centered care. Satisfaction in healthcare reflects whether a patient’s expectations for a consultation were met, influencing outcomes like reliance on services and treatment compliance (from the National Library of Medicine). Use and intention to use refer to a patient’s current use and future willingness to engage with services or technology, indicating adoption patterns. Attitude represents an individual’s fixed positive or negative evaluation of an aspect of care. Patient experience includes all interactions within the healthcare system, such as care from HCPs and staff, and encompasses factors like prompt appointments and effective communication, which are vital for healthcare quality [17]. Lastly, preferences refer to the ranking of alternatives based on relative value, helping determine the optimal choice in healthcare decisions. Preferences relate to an individual’s evaluation, often based on practical reasoning.

### Selection process and data extraction

Six reviewers (three senior researchers with a PhD, two PhD candidates, and one research assistant with a master’s degree) were involved in the selection process. At each stage, at least two reviewers per article were involved and worked independently. Conflicts were managed by senior researchers. We used the review management tool “Covidence” to ensure a standardized process. References from included studies were screened to find potential additional studies.

The extraction process was conducted independently by two PhD candidates or by a PhD candidate and a research assistant. A total of 30% of the studies were assessed by the three other researchers. Several rounds were conducted by authors until a convergence of the results was achieved.

The data extraction process followed the PICOS framework, namely Population, Intervention, Comparator, Outcome, and Setting. We only restricted the population to adults, as all individuals are likely to be patients. In addition to patients, we also included caregivers. Phone-based and video-based remote consultations were considered as the intervention modality which we compared to usual face-to-face consultation or with no comparator. Satisfaction, use and intention to use, attitudes, experiences, and preferences were retained as outcomes. Finally, we retained qualitative, quantitative, and mixed methods study designs.

All studies were synthesized in a single table (Table 2) where first author name, country and year of the study, research approach, target population, sampling method, number of observations (with completion rate, percentage of females, and mean age if mentioned in the study), and type of consultation were reported. For quantitative studies, quantitative approach, quantitative data analysis, and variables of interest were reported. For qualitative studies, qualitative approach, qualitative research method, and qualitative data analysis were reported. For mixed-method studies, quantitative and qualitative data were reported as well as mixed method design. Several rounds between the coauthors were performed to categorize levers and barriers to teleconsultation until themes and sub-themes emerged from the data extraction.

**Table 2:** Studies’ details

First author, publication year	Country	Year of the study^a^	Research approach	Target population	Sampling method	Nb. Observations (completion rate, nb. women, mean age [± SD])	Type of consultation	Quantitative approach	Quantitative data analysis	Dependent variable	Qualitative approach	Qualitative research method	Qualitative data analysis	Mixed method design
Abraham, 2022 [32]	U.S.	2020	Quantitative	Patients in general, urban area	Convenience sampling	79 (47%, 66%, n.r.)	Video/Phone	Survey	Bivariate	n.a.	n.a.	n.a.	n.a.	n.a.
Adams, 2023 [21]	Canada	2020	Qualitative	Socially vulnerable patients	Convenience sampling	29 (64%, 48%, n.r.)	Video/Phone	n.a.	n.a.	n.a.	Qualitative description	In-depth interviews	Framework analysis	n.a.
Aghajafari, 2022 [22]	Canada	n.r.	Qualitative	Elderly patients (> 55), urban area	Convenience sampling	29 (n.r., 17, 68 [n.r.])	Phone	n.a.	n.a.	n.a.	Qualitative description	Semi-structured interviews	Thematic analysis	n.a.
Anderson, 2021 [55]	U.K.	2021	Qualitative	Patients in general	Convenience sampling	44 (n.r.)	Video/Phone	n.a.	n.a.	n.a.	Qualitative description	Open-ended questions	Color coding (n.r.)	n.a.
Assing Hvidt, 2022 [43]	Denmark	2020	Qualitative	Patients in general	Convenience sampling	27 (n.r., 15, 48.67 [± 15.53])	Video	n.a.	n.a.	n.a.	Qualitative description	Semi-structured interviews	Thematic analysis	n.a.
Atherton, 2018 [23]	U.K.	2015	Qualitative	“Hard to reach” patients	Purposive sampling	39 (n.r., 25, n.r.)	Video/Phone	n.a.	n.a.	n.a.	Ethnography	Interviews	One-sheet-of-paper mind-map method	n.a.
Bali, 2007 [33]	India	2005	Qualitative	Patients in general, rural area	Convenience sampling	387 (93%, n.r., n.r.)	Phone	n.a.	n.a.	n.a.	Qualitative description	Records of phone calls	Counting of benefits	n.a.
Ball, 2018 [44]	U.K.	n.r.	Qualitative	Patients and carers	Purposive sampling	43 (n.r., 30, n.r.)	Phone	n.a.	n.a.	n.a.	Qualitative description	Semi-structured interviews	Thematic analysis	n.a.
Bhatia, 2022 [24]	U.S.	2020	Mixed	Elderly patients (≥ 65)	Convenience sampling	208 (84.6%, 61.5%, 74.4 [± 4.4])	Video/Phone	Survey	Multivariate	Overall satisfaction with TC	Qualitative description	Open-ended questions	Thematic analysis	Convergent design
Binder-Olibrowska, 2022 [26]	Poland	2021	Mixed	Patients with specific medical condition	Convenience and snowball sampling	219 (n.r., 126, 50.74 [± 17.16])	Video/Phone	Survey	Multivariate	Interest in TC	Qualitative description	Open-ended questions	Content analysis	Convergent design
Bittleston, 2022 [27]	Australia	2020	Qualitative	Patients with specific medical condition	Voluntary sampling	72 (n.a., 63, n.r.)	Phone	n.a.	n.a.	n.a.	Qualitative description	Open-ended questions	Content analysis	n.a.
Brown, 1995 [58]	U.K.	1992	Quantitative	Patients in general	Convenience sampling	Control group: 134 (51.74%, 70.1%, 52.8 [n.r.]) Phone consulters: 241 (93.05%, 71.4, 48.7 [n.r.])	Phone	Cross-sectional analytic study	Bivariate	n.a.	n.a.	n.a.	n.a.	n.a.
Buchanan, 2021 [40]	U.K.	2018	Quantitative	General population	Quota sampling by gender, age, ethnicity and geographic region	734 (74%, 375, 47 [± 17])	Video/Phone	Survey	Multivariate	Choice between F2F and TC	n.a.	n.a.	n.a.	n.a.
Burton, 2022 [34]	Canada	2020	Qualitative	Patients in general, rural area	Convenience sampling	8 (n.r., 6, 60.5 [median])	Video/Phone	n.a.	n.a.	n.a.	Qualitative description	Focus groups	Thematic analysis	n.a.
Chen, 2022 [35]	U.S.	2020	Quantitative	Patients in general, urban area	Convenience sampling (EHR data)	133,830 telehealth users (n.a., 82,997, n.r.) 91,317 telehealth non-users (n.a., 52,356, n.r.)	Video/Phone	Cross-sectional analytic study	Multivariate	Number of primary care visits by telehealth user status	n.a.	n.a.	n.a.	n.a.
Chudner, 2019a [64]	Israel	n.r.	Qualitative	Patients in general	Convenience sampling	42 for interviews (n.r., 28, 38.8 [n.r.]) 30 for attribute ranking exercise (n.r., 23, 39.6 [n.r.])	Video	n.a.	n.a.	n.a.	Narrative research	Semi-structured focus groups	Content analysis	n.a.
Chudner, 2019b [59]	Israel	n.r.	Quantitative	Patients in general	Web-based panel	508 (n.r., 281, 40.4 [± 14.3])	Video	Survey	Multivariate	Choice between F2F and TC	n.a.	n.a.	n.a.	n.a.
Ciecko, 2023 [50]	Poland	2021	Quantitative	Patients in general	n.r.	408 (n.r., 327, 35.52 [± 14.61])	Phone	Survey	Bivariate	n.a.	n.a.	n.a.	n.a.	n.a.
Curtis, 2021 [36]	New Zealand	2020	Mixed	Patients in general, urban area	Convenience sampling	108 (42%, 72, 54 [median])	Phone	Survey	Multivariate	Satisfaction with PC Likelihood to recommend PC to a friend	Qualitative description	Open-ended questions	Inductive thematic analysis	Convergent design
Devillers, 2023 [56]	France	2021	Quantitative	Patients and carers	Convenience and snowball sampling	307 (95.23%, 208, 43.1 [± 13.7])	Video/Phone	Survey	Bivariate	n.a.	n.a.	n.a.	n.a.	n.a.
Dixon, 2008 [41]	U.S.	n.r.	Quantitative	Patients in general	Convenience sampling	30 (n.r.)	Video	Randomized crossover design	Bivariate	n.a.	n.a.	n.a.	n.a.	n.a.
Dixon, 2009 [37]	U.S.	2007	Quantitative	Patients in general, urban area	Convenience sampling	152 (n.r.)	Video	Randomized crossover design	Bivariate	n.a.	n.a.	n.a.	n.a.	n.a.
Donaghy, 2019 [45]	U.K.	n.r.	Qualitative	Patients in general	Convenience sampling	21 (46.67%, 10, 45.24 [± 16.43])	Video/Phone	n.a.	n.a.	n.a.	Qualitative description	Semi-structured interviews	Thematic analysis	n.a.
Donaghy, 2023 [25]	U.K.	2019	Mixed	Elderly patients	Convenience sampling	165 (63%, 106, n.r.)	Video/Phone	Survey	Bivariate	n.a.	Qualitative description	Open-ended questions	Thematic analysis	Convergent design
Duncan, 2021 [57]	U.K.	2020	Qualitative	Patients in general	Convenience and voluntary sampling	Survey A: 150 (n.a., n.r., n.r.) Survey B: 56 (79%, n.r., n.r.)	Video/Phone	n.a.	n.a.	n.a.	Qualitative description	Open-ended questions	Narrative analysis	n.a.
Esber, 2023 [28]	Germany	2020	Quantitative	Patients with specific medical condition	Convenience sampling	371 (89.47%, 191, 42.28 [± 16.09])	Video	Survey	Multivariate	Acceptance of VC	n.a.	n.a.	n.a.	n.a.
Garrett, 2022 [46]	New Zealand	2021	Qualitative	Patients aged between 15 and 25	Convenience and voluntary sampling	132 (38%, 90, n.r.)	Video/Phone	n.a.	n.a.	n.a.	Qualitative description	Open-ended questions	Template analysis	n.a.
Greenhalgh, 2022 [53]	U.K.	2019	Qualitative	Patients in general	Convenience sampling	33 (n.r., n.r., n.r.)	Video/Phone	n.a.	n.a.	n.a.	Qualitative description	Interviews and focus groups	Planning and Evaluating Remote Consultation Services (PERCS)	n.a.
Han, 2022 [38]	Canada	n.r.	Qualitative	Patients in general, urban area	Voluntary and snowball sampling	21 (n.a., 16, 36.73 [± 15.57])	Video	n.a.	n.a.	n.a.	Grounded theory	Semi-structured and scenario-based interviews	Open, axial, and selective coding methods	n.a.
Javanparast, 2021a [29]	Australia	2020	Qualitative	Patients with specific medical condition	Convenience sampling	30 (99.91%, 17, n.r.)	Phone	n.a.	n.a.	n.a.	Qualitative description	Semi-structured interviews	Inductive and deductive thematic analysis	n.a.
Javanparast, 2021b [30]	Australia	2020	Qualitative	Patients with specific medical condition	Convenience sampling	30 (99.91%, 17, n.r.)	Phone	n.a.	n.a.	n.a.	Qualitative description	Semi-structured interviews	Inductive and deductive thematic analysis	n.a.
Kludacz-Alessandri, 2021 [60]	Poland	2021	Quantitative	Patients in general	Convenience sampling	99 (94%, 56%, 55 [n.r.])	Phone	Survey	Multivariate	Quality of consultation and communication with GP	n.a.	n.a.	n.a.	n.a.
Kowalski, 2018 [61]	U.S.	n.r.	Quantitative	Patients in general	Convenience sampling	233 (n.r., 38.2%, 52.7 [± 17])	Video/Phone	Survey	Bivariate	n.a.	n.a.	n.a.	n.a.	n.a.
Leng, 2016 [47]	U.K.	n.r.	Mixed	Patients in general	Convenience sampling	270 (91.2%, 162, n.r.)	Video	Survey	Bivariate	n.a.	Qualitative description	Open-ended questions	Thematic coding frame	Convergent design
Mangalji, 2022 [66]	Canada	2020	Quantitative	Patients in general	Convenience sampling	426 (54.8%, 65.4%, 61.4 [± 16.5])	Phone	Survey	Bivariate	n.a.	n.a.	n.a.	n.a.	n.a.
Manski-Nankervis, 2022 [65]	Australia	2020	Quantitative	Patients in general	Convenience sampling	499 (9.8%, 347, 31.8 [± 11.40])	Video	Survey	Multivariate	Perceptions of VC	n.a.	n.a.	n.a.	n.a.
Mathew, 2021 [63]	Australia	2020	Quantitative	Patients in general	Convenience and voluntary sampling	154 (60.65%, 105, n.r.)	Video/Phone	Survey	Multivariate	Likelihood of attending VC	n.a.	n.a.	n.a.	n.a.
McKinstry, 2009 [48]	U.K.	n.r.	Qualitative	Patients in general	Purposive sampling	33 (16,75%, 20, n.r.)	Phone	n.a.	n.a.	n.a.	Qualitative description	Focus groups	Framework analysis	n.a.
McKinstry, 2010 [49]	U.K.	n.r.	Quantitative	Patients in general	Convenience sampling	Phone-consultation: 47 (n.r., 28, n.r.) Face-to-face consultation: 59 (n.r., 38, n.r.)	Phone	Survey	Multivariate	Communication behaviors (Roter Interaction Analysis Scale (RIAS))	n.a.	n.a.	n.a.	n.a.
Mohan, 2022 [42]	U.S.	2020	Quantitative	Patients in general	Convenience sampling	797 (26.7%, 591, 48.70 [± 17.67])	Phone	Survey	Bivariate	n.a.	n.a.	n.a.	n.a.	n.a.
Payne, 2001 [54]	U.K.	n.r.	Qualitative	Patients in general	Convenience sampling	47 (50%, 23, n.r.)	Phone	n.a.	n.a.	n.a.	Qualitative description	Semi-structured interviews	Framework analysis	n.a.
Poitras, 2022 [31]	Canada	2020	Qualitative	Patients with specific medical condition	Convenience sampling	39 (79.59%, 23, 60.5 [n.r.])	Video/Phone	n.a.	n.a.	n.a.	Qualitative description	Semi-structured interviews	Deductive thematic analysis	n.a.
Powell, 2017 [51]	U.S.	2015	Qualitative	Patients in general	Convenience sampling	19 (59.38%, 53%, 43 [median])	Video	n.a.	n.a.	n.a.	Qualitative description	Semi-structured interviews	Content analysis	n.a.
Reed, 2020 [52]	U.S.	2016	Quantitative	Patients in general	Convenience sampling (EHR data)	1,131,722 patients (n.a., 54.86%, n.r.)	Video/Phone	Cross-sectional analytic study	Multivariate	Choice of consultation modality	n.a.	n.a.	n.a.	n.a.
Rose, 2021 [39]	U.S.	2017	Mixed	Patients in general, urban area	Convenience sampling	426 (65.7%, 273, 46 [± 15.5])	Video	Survey	Multivariate	Overall satisfaction, patient-clinician engagement, comfort and ease with virtual technology	Narrative research	Open-ended questions	Framework analysis	Convergent design
von Weinrich, 2022 [62]	Germany	2020	Quantitative	Patients in general	Quota sampling by age and gender	350 (5.7%, 52.6%, n.r.)	Video	Survey	Multivariate	Choice between F2F and TC	n.a.	n.a.	n.a.	n.a.

^a^If the study took place over several years, the year of launch of the study is reported

* n.a *non-applicable, *n.r *not reported, *EHR *Electronic health records, *GP *General practitioner, *TC *Teleconsultation, *PC *Phone consultation, *VC *Video consultation, *U.S *United-States, *U.K *United-Kingdom

All results related to our study outcomes were reported in a Microsoft Excel^®^ file. For quantitative results, the sign of the relationship between the outcomes and the determinants of synchronous teleconsultation were retrieved, as well as their significance level (**** *p* <.001, *** *p* <.01, ** *p* <.05, *p <.1, n.s. [not significant]). For qualitative results, relevant quotes about the relationship between the outcomes and the determinants of synchronous teleconsultation were reported. Because of the diverging types of articles retained for this systematic review, no unique effect measure was retained. All elements were finally binary coded to get a 0/1 presentation (1 if the study mentioned the determinant, 0 otherwise). For mixed-method studies (*n* = 6), a determinant wax coded as 1 if it appeared in the qualitative part, the quantitative part, or both, i.e., no weighting was applied for those studies. See Additional file 3 for complete data extraction. 

### Quality assessment

As we considered qualitative, quantitative, and mixed method studies, we used the second version of the Mixed Methods Appraisal Tool (MMAT) to assess the quality of studies [18]. Two coauthors assessed the quality of studies independently before results were compared. Another coauthor resolved the divergences. Assessment was automated in a Microsoft Excel^®^ file. The results are provided in Additional file 4 as defined by the authors of the tool. To sum-up the quality of included studies, we coded the Yes/No/Can’t tell modalities 1, −1, and 0, respectively, and we computed an average for each study to get a quality score (ranging from − 1 to 1). As mentioned by the authors of the MMAT, comparing the quality of studies based on a score is discouraged. Thus, scores are given solely for the purpose of making reading easier.

## Results

### Studies details

**Fig. 1:**
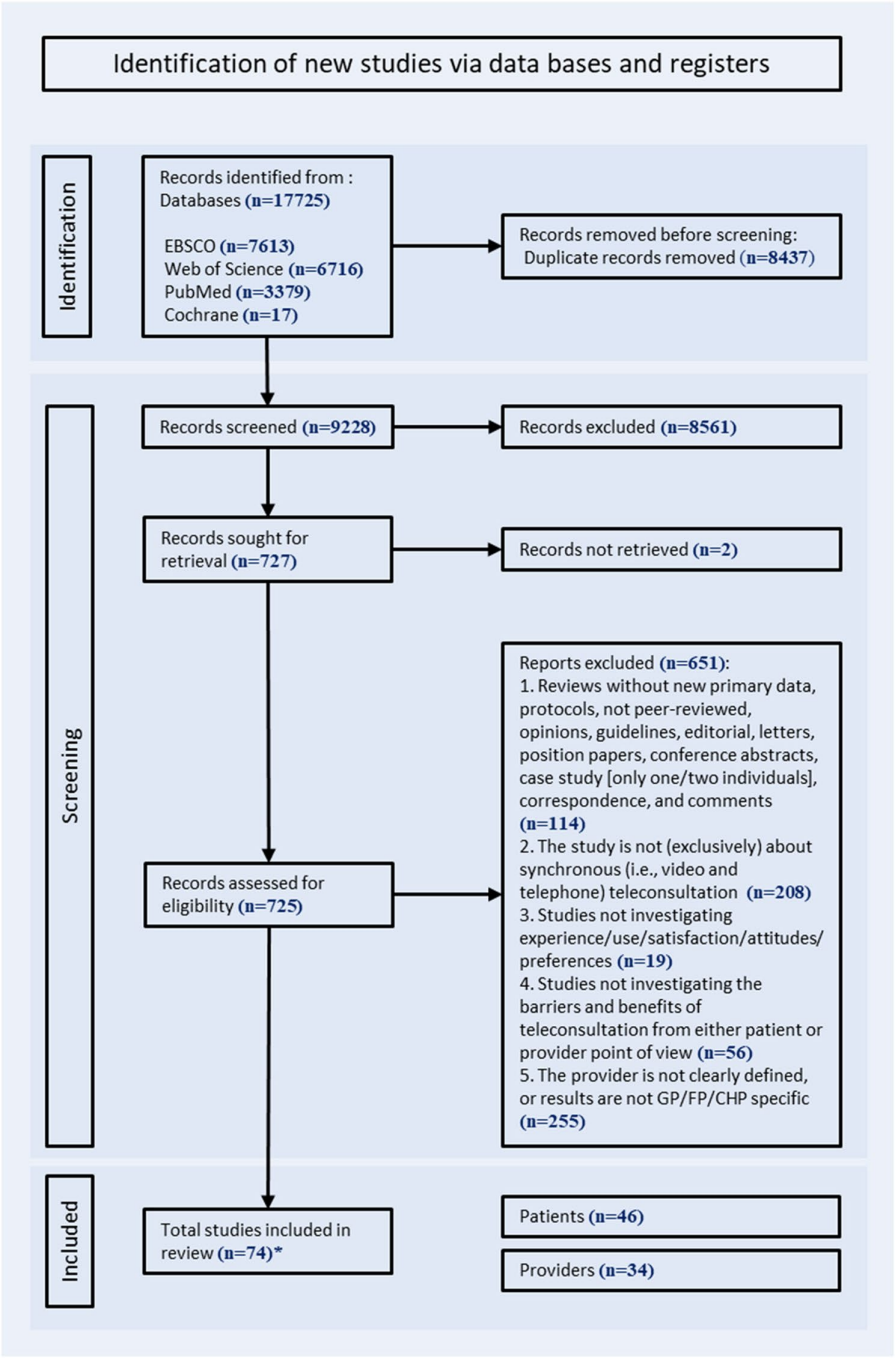
Flowchart of studies selection process on March 23, 2023 *Several studies were about both patients and general practitioners

**Fig. 2:**
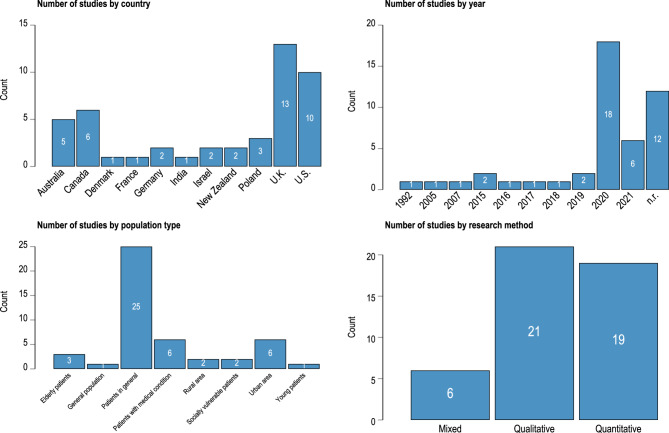
Country, year, population type, and research method of studies. Note: n.r., not reported

A total of 17,725 studies were imported from the databases, of which 8,437 were duplicates. Among the 9,288 studies screened on abstracts, 8,561 were excluded, thus resulting in a total of 727 studies eligible (including 2 studies that could not be retrieved [19, 20]. The screening of full texts resulted in 74 studies included for the systematic review of which 46 were about patients. No study appeared to meet the inclusion criteria but was excluded. Study details are reported according to the PICOS framework (Table 2). The selection process is presented in the flowchart (Fig. 1). Nineteen studies were quantitative, 21 were qualitative, and 6 were mixed-method studies (Table 2; Fig. 2).

#### Studies years and locations

Most studies were conducted in Northern Europe (*n* = 14), Northern America (*n* = 16), and Australia or New Zealand (*n* = 7). A few others were conducted elsewhere in Europe (*n* = 6) and Southern or Western Asia (*n* = 3) (Table 2; Fig. 2). Most studies were conducted after 2019 (*n* = 36) and 12 studies did not report the year of the conduct of the study (Table 2; Fig. 2).

#### Studies populations

Most studies used convenience or purposive sampling in restricted populations, e.g., from health facilities, or inpatients or outpatients’ services (Table 2). Twenty-five studies did not consider any specific population (Table 2; Fig. 2). Two studies implied socially vulnerable populations, four studies implied elderly or young populations [21–25], six studies implied patients with specific medical conditions [26–31], eight studies were specific to either urban or rural [22, 32–39], and one study focused on the general population [40] (Table 2).

Because of the diversity of methods adopted, sample sizes were highly heterogeneous. For quantitative studies, it ranged from 30 participants [41] to 797 participants [42] (excluding studies using electronic records, *n* = 2). For qualitative studies, it ranged from 8 participants [34] to 387 participants [33] (Table 2). Nineteen studies focused on both video and phone consultations, twelve were about video consultations, and fifteen were about phone consultations (Table 2).

### Quality assessment

The global average quality score was equal to 0.51 (ranging from − 1 to 1). Quality scores were equal to 0.69, 0.28, and 0.51 for qualitative, quantitative, and mixed method studies, respectively (Table 3).

**Table 3:** Quality assessment (Mixed methods appraisal Tool)

ID	Qualitative studies	Quantitative studies	Mixed method studies
Abraham, 2022 [32]	-	−0.60	-
Adams, 2023 [21]	1.00	-	-
Aghajafari, 2022 [22]	1.00	-	-
Anderson, 2021 [55]	−0.60	-	-
Assing Hvidt, 2022 [43]	1.00	-	-
Atherton, 2018 [23]	0.20	-	-
Bali, 2007 [33]	−0.60	-	-
Ball, 2018 (Ball et al., 2018)	0.80	-	-
Bhatia, 2022 [24]	1.00	-	0.87
Binder-Olibrowska, 2022 [26]	0.20	-	0.27
Bittleston, 2022 [27]	0.40	-	-
Brown, 1995 [58]	-	−0.40	-
Buchanan, 2021 [40]	-	1.00	-
Burton, 2022 [34]	0.80	-	-
Chen, 2022 [35]	-	0.20	-
Chudner, 2019b [59]	-	1.00	-
Chudner, 2019a [64]	1.00	-	-
Ciecko, 2023 [50]	-	−0.60	-
Curtis, 2021 [36]	-	-	0.67
Devillers, 2023 [56]	-	−0.20	-
Dixon, 2009 [37]	-	−0.40	-
Dixon, 2008 [41]	-	0.00	-
Donaghy, 2019 [45]	1.00	-	-
Donaghy, 2023 [25]	-	-	0.33
Han, 2022 [38]	1.00	-	-
Duncan, 2021 [57]	0.60	-	-
Esber, 2023 [28]	-	1.00	-
Garrett, 2022 [46]	1.00	-	-
Greenhalgh, 2022 [53]	1.00	-	-
Javanparast, 2021a [29]	1.00	-	-
Javanparast, 2021b [30]	1.00	-	-
Kludacz-Alessandri, 2021 [60]	-	0.80	-
Kowalski, 2018 [61]	-	0.00	-
Leng, 2016 [47]	0.20	0.20	0.13
Mangalji, 2022 [66]	-	0.20	-
Manski-Nankervis, 2022 [65]	-	0.60	-
Mathew, 2021 [63]	-	0.80	-
McKinstry, 2010 [49]	-	0.60	-
McKinstry, 2009 [48]	1.00	-	-
Mohan, 2022 [42]	-	0.20	-
Payne, 2001 [54]	0.60	-	-
Poitras, 2022 [31]	1.00	-	-
Powell, 2017 [51]	1.00	-	-
Reed, 2020 [52]	-	0.60	
Rose, 2021 [39]	-	-	0.80
von Weinrich, 2022 [62]	-	0.60	-
Average	0.68	0.28	0.51
Total average	0.50		

Score ranged from − 1 (low quality) to 1 (high quality)

### Themes and sub-themes

Most studies were practice-centered, and results were intrinsically context-specific, but most were convergent. Five themes were defined according to a consensus between coauthors that emerged from several rounds. The number of studies included in each theme and sub-theme are presented in Table 4.

**Table 4:** Number of studies by themes and sub-themes

Themes and Sub-themes	Number of studies (*n* = 46)
Consultation-related	
Teleconsultation purpose	23 (50.00%)
Health issues	19 (41.30%)
Administrative and documentation	10 (21.74%)
Assessment and guidance	15 (32.61%
Teleconsultation setting	18 (39.13%)
Quality of care	27 (58.70%)
Teleconsultation undermines the accuracy/reliability	19 (41.30%)
Duration of consultation	14 (30.43%)
Teleconsultation safety	2 (4.35%)
Role of consultation by phone or video as a modality of consultation	13 (28.26%)
Technology-related	17 (36.96%)
Provider-related	4 (8.70%)
Patient-related	
Patient’s socioeconomics and demographics characteristics	34 (73.91%)
Patient’s socioeconomic profile	12 (26.09%)
Patient’s demographic profile	27 (58.70%)
Patient’s medical condition	21 (45.65%)
Patient’s abilities, experience, and attitudes towards teleconsultation	32 (69.57%)
Convenience for the patient	31 (67.39%)
Time-related	29 (63.04%)
Patient-centered care	32 (69.57%)
Institution-related	24 (52.17%)

#### Consultation-related

The first theme explored elements surrounding the consultation itself, namely: (1) The consultation purpose (50% of studies), (2) The consultation setting (39.13% of studies), (3) The quality of care (58.70% of studies), (4) The role of teleconsultation as a modality of care delivery (28.26% of studies), and (5) Technology-related factors (36.96% of studies).

*Teleconsultation purpose.* Teleconsultation purposes were the most extensively examined element in the literature, encompassing various contexts such as health-related, administrative, documentation-related, and assessment and referral-related purposes (Additional file 5). Health-related consultations could be categorized by health issues, complexity, urgency and severity, and the disease’s time course (e.g., routine/follow-up consultation, first visit). Mental health and dermatological issues were judged as most suitable for teleconsultation [21, 29, 31, 36, 38, 43–48], followed by sensitive or personal issues [22, 31, 36, 38, 45–47]. Minor or common health issues, and straightforward concerns were identified as best suited for teleconsultation [21, 24, 26, 27, 29, 36, 38, 42–44, 46–48], as well as routine and follow-up consultations [24, 30, 31, 33, 43, 45, 47–49] in contrary to new health issues [23, 49]. Administrative and documentation-related consultations were primarily focused on prescription renewals [21, 27, 29–31, 42, 46, 47, 50]. Assessment and guidance-related consultations predominantly involved requests for laboratory tests or medical imaging as well as discussions around laboratory or imagery results [29–31, 42, 43, 45–48] or discussions around treatments [26, 33, 36, 45]. Both were considered as well-suited for teleconsultation. Although there is a general trend about which purpose is well-suited for a teleconsultation, some disagreements were underlined. For instance, some patients expressed a need for a physical examination [21, 36, 43, 47, 48, 51] even if their purpose did not rely on a health issue. Others expressed different opinions related to personal or sensitive health issues [22, 36, 38, 45–47].

*Teleconsultation setting.* Teleconsultation setting enlarges features such as a virtual waiting room or dedicated platforms with possibilities to share information (Additional file 6). Among barriers, patients expressed fear to miss their appointment or to be missed by their GP because of the setting of the virtual waiting room [21, 24, 43, 45]. Involving a third party as a family member was perceived whether as an ease or as a benefit, in particular, the help that an elderly’s family member can provide in carrying out the teleconsultation [22, 24, 25, 28, 34, 52]. The ease of appointment booking is another important benefit of teleconsultation. Indeed, it allows patients to book an appointment early and at a precise time whether directly from the clinical office or by a dedicated platform [21, 26, 27, 34, 39, 44].

*Quality of care*. A low perceived quality of care is an important barrier to teleconsultation (Additional file 7). Lack of or difficulties in physical examination [21, 24, 26, 31, 38, 42, 46, 48, 53, 54]; lack of visual, non-verbal, behavioral cues, and intimacy [21, 24, 26, 27, 29, 36, 38, 43, 44, 47, 48, 55]; and a higher risk of error in the diagnostic or misunderstanding between the patient and the practitioner [21, 24, 26, 44, 46–48, 54, 55] were all perceived as barriers. Although it is possible to perform a physical examination by videoconference following GP’s indications, it is generally perceived to be of low value for patients [21, 24, 26, 31, 38, 42, 46, 48, 53, 54] who perceived a risk of misunderstanding, misinterpretation, and misdiagnosis [21, 24, 26, 44, 46–48, 54, 55]. Moreover, some patients felt unable to describe their symptoms by phone or video. Lack of visual, non-verbal, and behavioral cues impaired the relationship with the GP and reinforced the feeling that the diagnostic may be wrong [21, 24, 27, 29, 36, 38, 43, 48]. Nevertheless, most patients expressed a preference for videoconference over phone consultation.

*Role of consultation by phone or video as a modality of consultation*. The role of teleconsultation alongside face-to-face consultation was highly discussed in the literature (Additional file 8). Teleconsultation was perceived as a complement or as a substitute to consultation [24, 26, 29, 30, 39, 43, 44, 54, 56, 57]. For instance, some studies mentioned that teleconsultation could serve as a triage tool and that patients often got a remote consultation to know if it is necessary to have a physical consultation. Other studies highlighted that, depending on the purpose, teleconsultation could be a perfect substitute (e.g., for prescription renewal). There is a high heterogeneity in patients’ perceptions of the role of teleconsultation compared to a face-to-face consultation. Nonetheless, studies showed that teleconsultation should not absolutely replace face-to-face consultation.

*Technology-related*. Three distinct points related to the technological aspects of teleconsultation have been identified (Additional file 8). First, technical issues are frequently mentioned as a significant barrier [24, 38, 39, 43, 45, 48, 56]. These issues primarily involve internet connection problems, which can result in low video and sound quality or disconnection from the consultation. In a video consultation setting, this may require switching to a phone consultation. Second, teleconsultations by video require appropriate equipment, such as a reliable internet connection, a computer with a microphone, or a smartphone [24, 31, 32, 44, 57, 58]. However, the literature indicates that there are substantial inequalities regarding income, knowledge, and technological abilities. Lastly, numerous studies highlighted concerns about privacy and confidentiality [38, 44, 47, 57]. Patients feared that someone might be in the room with the practitioner without being visible or that the practitioner might record the audio and/or video without their consent. On the patient’s side, some participants worried about their privacy at home, for instance, that a family member could overhear their conversation [21, 27, 45, 46].

#### Provider-related

The second theme is about providers (8.70% of studies). Patients’ perceptions of their providers’ use of teleconsultation have received very little attention in the literature (Additional file 9). Only a few elements have been identified, notably the provider’s communication skills, empathy and punctuality [31, 39, 54]. A study by Buchanan et al. (2021) [40] looked at the effect of a doctor’s reputation on the choice of teleconsultation. 

#### Patient-related

The third theme related to patients’ characteristics, namely, (1) Socioeconomics and demographics characteristics (73.91% of studies), (2) Medical condition (45.65% of studies), (3) Abilities, experiences, and attitudes (69.57% of studies), as well as (4) The benefits they get from teleconsultation (67.39% of studies).

*Patient’s socioeconomics and demographics characteristics*. Patient’s socioeconomics and demographics profile was highly discussed in the literature (Additional file 10). Female, young, workers, and individuals having family (e.g., parents with young children) were cited as the most well-suited to teleconsultation, whereas elderly and individuals with a foreign language were cited as the least well-suited [22, 23, 26, 28, 29, 31, 33, 35, 36, 38, 39, 42–48, 52, 55, 56, 58–61].

*Patient’s medical condition*. Individuals with mental health problems were cited as the most well-suited to teleconsultation [21, 28, 38, 44, 53, 57], in contrast to individuals with hearing or visual problems [21, 25, 26, 31, 34, 36, 43, 46, 57] (Additional file 10). Other health issues judged suitable for teleconsultation were related to chronic conditions, disabilities and restricted mobility [24, 26, 35, 44, 46–48]. Only few studies focused on patients with specific medical conditions [26–31], suitability for teleconsultation of those patients as reflected in the literature is then not representative of them.

*Patient’s abilities*,* experience*,* and attitudes towards teleconsultation*. In general, patients exhibited a preference for face-to-face consultations over remote consultations and for video consultations over phone consultations, even among those who had never experienced teleconsultation [22–25, 29, 31, 40, 42, 43, 45, 47, 53, 55, 57, 59, 61, 62] (Additional file 11). A positive prior experience with teleconsultation was also identified as a strong predictor of future use [34, 35, 42, 55, 56, 58, 60, 63]. Additionally, patients’ general or technology-related knowledge and abilities, such as their proficiency in communicating using technology, were found to be significant factors influencing the use of teleconsultation [22, 24, 26, 28, 29, 32, 34, 44–47, 50, 52, 55, 57–59], especially in the elderly population.

*Convenience for the patient*. Time-related factors were widely studied (Additional file 12). Three components can be defined. First, teleconsultation overcomes distances, saving travel time and costs [22, 24, 26, 27, 29, 31, 33, 34, 36, 44, 45, 47, 48, 52, 55, 64, 65]. Second, waiting times before the next available appointment [24, 26, 29, 38, 40, 44, 45, 47, 59, 60, 62, 64] and on the appointment day [22, 24, 26, 33, 34, 36, 38, 44–46, 51, 59, 62, 64, 66] were considered shorter than face-to-face consultations, facilitating teleconsultation. Few studies found longer waiting times for teleconsultations [26, 27, 54]. Lastly, scheduling flexibility allowed those with constraints, like workers or parents, to book at their convenience [27, 45, 47]. For instance, teleconsultation avoided taking half a day off and enabled scheduling during breaks or continuing work. Although context-dependent, one study found the lack of a specific time for a phone consultation inconvenient [36]. Teleconsultation allowed patients not to go to a clinic [39, 47, 55] and to stay home when ill or create a “safe place” for consultations [27, 46]. However, some patients faced challenges finding an appropriate or private location, such as being in a store or on public transport [46, 53]. Teleconsultation also avoided traveling in inclement weather [24, 47, 64]. Some patients reported that teleconsultation either increased anxiety, such as for parents or when setting up the consultation [27, 48], or decreased it, by avoiding public transport or crowded waiting rooms [24, 33, 34, 37, 38]. 

#### Patient-centered care

The fourth theme deals with patient-centered care (Additional file 13) (69.57% of studies). Patient-centered care is a healthcare model focusing not only on patients’ symptoms and medical history but also on their emotional and social components, values, and preferences (from the National Library of Medicine^)^. Patients and their family play an active role in decision-making about the patient’s care. Relationships between patients and providers, involvement of both parties, and better management of patient’s healthcare pathway (i.e., continuity of healthcare) and health issues were all elements discussed. The most cited element fostering the use of teleconsultation was the preexisting relationship between patients and providers [21–24, 26–29, 36, 39, 43–45, 48, 52, 59, 62, 64]. Patients expressed a need to already know the general provider before getting a teleconsultation. One study found that not knowing the GP was an issue for patients [28]. Literature showed different results about management of a patient’s health needs (i.e., if his/her concerns and worries were well addressed). Some patients expressed a desire that the consultation should be fast and focused on a single problem [21, 27, 29, 39, 50]. While teleconsultation seemed to be well-suited for them, other patients complained that it did not allow them to express all their needs [31, 48, 49]. Concerning continuity of care, results are quite divergent. For some patients, teleconsultation allows to maintain their relationship with their practitioner or to access healthcare [21, 29, 31, 33, 44, 48] while for some other patients, it impaired their healthcare pathway because of a lack of follow-up [25, 57]. Continuity of care is a matter of a preexisting relationship with the practitioner and of good communication between patients and practitioners, explaining the divergent results. Finally, providers’ involvement (e.g., explanation, attention/focus) is also an important element [25, 26, 39, 41, 49]. Patients expressed different opinions on the fact that GPs were not as focused on teleconsultation as they would be for a face-to-face consultation. For some, teleconsultation allows GPs to focus and maintain attention on a single problem [24, 43, 59, 64] while other patients reported that GPs were focused on their computer or that they could not say if they looked at them [27, 38, 54].

#### Institution-related

The fifth and last theme deals with institutional-related factors and is mostly about access to care and teleconsultation information dissemination (Additional file 14) (52.17% of studies). Financial barriers of access to care were widely studied such as costs for patients, direct or indirect [31, 33, 39, 40, 46, 47, 52]. Generally, teleconsultation was perceived cheaper by patients than face-to-face consultation as it eliminates travel costs [47, 64]. Patients were not willing to pay more for a teleconsultation compared to a traditional face-to-face consultation and expressed a fear of an additional copayment [29, 42]. One study also highlighted that patients expressed dissatisfaction when a face-to-face visit is necessary after a teleconsultation as they must pay for both consultations [27]. Reimbursement by health insurance is only discussed in one study [39]. Literature reported that patients are often not well-informed about the availability of and the functioning of teleconsultation into the healthcare system (e.g., which modalities are available, how they work), thus limiting patients’ acceptation of teleconsultation [21, 23, 39, 44, 53, 58]. Access to prescription and medication were also discussed. In some contexts, teleconsultation made easier the access to medication or prescription retrieval [26, 40, 44] whereas e-prescriptions were perceived as a barrier in some studies due to technical difficulties [39, 48]. Some studies reported exclusion based on income, digital skills and ease with technology, or related to other sociodemographic characteristics [33, 34, 43, 44]. Only two studies reported that teleconsultation could be digitally exclusive, especially for elderly, housebound, or people with limited technical abilities [34, 43].

## Discussion

### Interpretation of the results in the context of existing literature

The findings of this systematic review align with previous literature. Consistent with Agbali et al. (2021) [8], patient satisfaction with teleconsultation was strongly influenced by perceived convenience and accessibility. Akesson et al. (2007) [9] similarly emphasized that telehealth technologies empower patients, enhancing their confidence and health outcomes through improved access to care. This review reaffirmed these insights, particularly for routine and follow-up consultations, where video consultations were preferred due to their visual interaction capabilities. Our results also aligned with Chen et al. (2022) [13], highlighting that video consultations were superior to phone calls for issues requiring visual assessments or nuanced communication, particularly in mental health. In contrast, phone consultations remained crucial for individuals with limited technological skills or inadequate internet access, as noted by Downes et al. (2017) [14]. Thiyagarajan et al. (2020) [15] further underscored the limitations of video consultations for complex or sensitive primary care issues, where face-to-face interactions were preferred. The results also supported Appleton et al. (2021) [10], noting telehealth’s role in maintaining mental health continuity during disruptions and identifying digital exclusion barriers that affect vulnerable populations. Bowman et al. (2023) [11] added to this understanding, demonstrating that videoconferencing interventions for COPD patients generally resulted in high patient satisfaction but were susceptible to technological challenges, which can impact the quality of care​.

### Strengths and limits of the systematic review

The first strength of this mixed-method systematic review lies in the diversity of sources used. References were extracted from four distinct databases (PubMed, Web of Science, EBSCO, and Cochrane Library). A second strength is the inclusion criteria; we did not limit the selection based on publication year, allowing us to trace the evolution of studies from the early 1990 s to present, with most studies published in 2020 and 2021 (24 studies out of 46, or 52.17%) during the COVID-19 crisis. We also did not restrict studies by country, highlighting the high prevalence of studies in some countries. Finally, the focus on synchronous teleconsultation with GPs avoids biases or specificities related to other specialties. Existing systematic reviews typically address multiple HCPs, specific health issues, or other consultation modalities. Lastly, this review incorporated data from quantitative, qualitative, and mixed-method studies, providing complementary evidence.

**Fig. 3:**
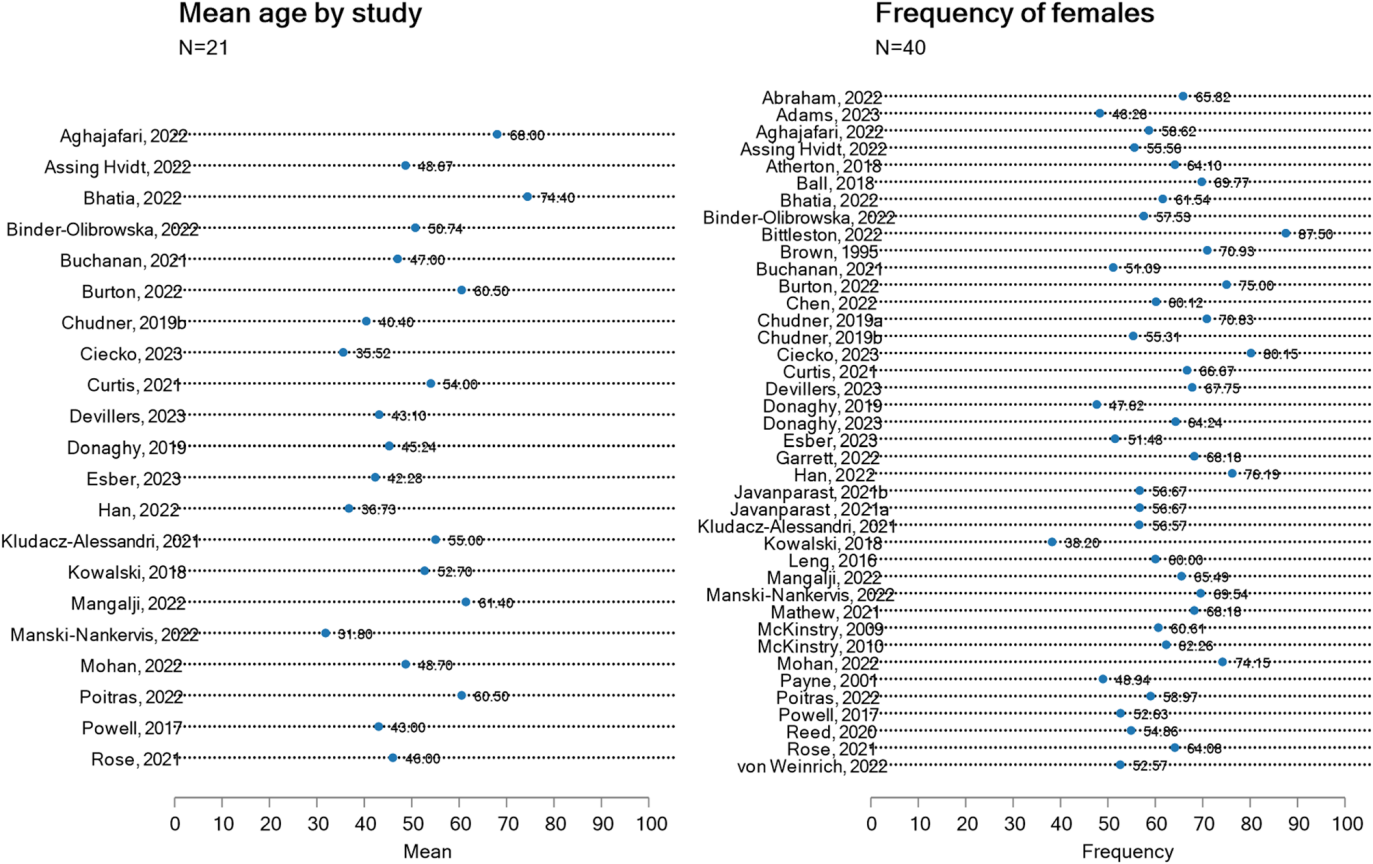
Mean age and frequency of females by study

The main limitation of the studies included in the review is their poor representativeness. First, only one study focused on the general population using a quota sampling method [40], while most other studies relied on convenience samples (38 studies out of 46, or 82.61%). Consequently, women and older individuals were largely overrepresented among respondents. Women indeed represented 61.9% of all respondents while the average age of respondents across studies was 49.8, far above most countries’ mean population age (Fig. 3). Moreover, studies from North America (United States and Canada, 16 studies out of 46, or 34.78%) and the United Kingdom (13 studies out of 46, or 28.26%) were overrepresented (Table 2; Fig. 2) and one study was conducted in a middle-income country, namely India [33]. The determinants of teleconsultation use also showed significant heterogeneity. The number of determinants ranged from 1 [66] to 38 [47], with an average of 12.24 determinants per study (Additional Files 15–17). Studies with higher proportions of female participants tended to include fewer determinants related to perceptions towards TC (e.g., consultation purpose, patients best suited for TC, time-related aspects), while studies involving older participants tended to include more determinants related to consultation purposes, time-related aspects, perceived accuracy and quality of care, as well as patients’ demographic, sociodemographic, and medical characteristics. Conversely, studies on older participants explored fewer determinants concerning the patient-GP relationship, technological aspects, and the cost of TC. Regional differences were also observed: studies from Australia and New Zealand (*n* = 7) more frequently addressed consultation purposes and patients’ demographic and sociodemographic characteristics, while studies from North America (16 studies out of 46, or 34.78%) and Europe (20 studies out of 46, or 43.48%) focused more on technological aspects of teleconsultation.

Because of the diversity of the research approaches employed in studies included in this mixed method systematic review, we did not perform a meta-analysis inherent to quantitative studies. Then, no sensitivity analysis, robustness checks, or subgroups analysis was performed.

### Policy implications and future research

Numerous guidelines are provided by countries that adopted teleconsultation. It is highly valuable to know if they are well suited according to patients’ preferences and if the actual use of teleconsultation respects these guidelines. We mainly explored French, Quebec (Canada), British, and United-States guidelines and we discussed here five points that emerged from these guidelines: (1) Hybrid care, (2) Consultation purpose, (3) Patients’ suitability, (4) Continuity of care, and (5) Privacy and confidentiality.

#### Hybrid care

Overall, the guidelines aligned with literature findings on patients’ preferences. Patients had straight preferences for video consultations over phone consultations, although in-person visits remained the preferred mode. According to the Health Resources and Services Administration from the U.S. Department of Health and Human Services, “*hybrid care*,* also called integrated care*,* combines in-person health care appointments with telehealth. This approach prioritizes the joint decision between the patient and their provider regarding whether and when a virtual visit is appropriate*.” Moreover, *“regular follow-up of patients must alternate between face-to-face consultations and teleconsultations*,* depending on the patient’s needs and the doctor’s assessment. Treating patients exclusively by teleconsultation undermines the ethical requirements of quality*,* safety*,* continuity of care and permanence of care.”* [6] Teleconsultation was mainly perceived as complementary to face-to-face consultation but not substitutable in the long run, except in few instances (e.g., prescription renewal). It should not be first proposed by healthcare providers. Instead, it should remain a choice for patients and no assumption about patients’ preferences for a modality should be stated by HCPs.

#### Consultation purpose

One of the issues of teleconsultation is the matter of the medical professional’s ability to establish a diagnosis in the absence of direct physical examination. The Quebec telehealth network provides a substantial list of clinical situations well-suited for teleconsultation [6]. “*In any case*,* the use of teleconsultation is a matter for the HCP to decide*,* and he or she must judge the relevance of remote rather than face-to-face medical care*.” [6] Regarding the most suitable consultation purposes for teleconsultation, the guidelines were again consistent with patients’ preferences expressed in the literature. Simple consultation purposes, such as requests for laboratory tests or prescription renewals, were most frequently cited as more suited for teleconsultation. The following were consultations for chronic disease management. Reasons related to information requests, obtaining certificates (e.g., sick leave), or referral letters, although simple, were infrequently cited in the literature. Mental and cognitive health issues were widely reported as suitable for teleconsultation, though these findings were typically reported by populations not directly affected by such issues. Finally, sensitive topics (e.g., sexuality), skin conditions, or issues requiring an in-depth physical examination were frequently deemed unsuitable for teleconsultation.

#### Patients’ suitability

Patients’ suitability for teleconsultation varies. The elderly were generally perceived as less suited for teleconsultation. Women, particularly mothers, were more inclined to use teleconsultation, likely due to caregiving responsibilities. Students and working individuals also favored teleconsultation, driven by busy schedules. Younger adults, more comfortable with technology, were seen as more likely to use teleconsultation. Few studies explored education and income levels, but higher educational attainment was associated with greater teleconsultation use. Teleconsultation was especially advantageous for patients with limited mobility, such as those in rural areas or with disabilities. However, visual and auditory impairments were significant barriers. Results regarding cognitive and mental health issues were mixed, as most respondents did not have these conditions themselves. Many findings focused on patients’ ability to conduct consultations. Patients lacking technological skills or equipment (e.g., microphone, camera, smartphone) or those uncomfortable with remote consultations were effectively excluded from teleconsultation use. These findings align with NHS England’s consideration of the general capacity required for teleconsultation, including cognitive, psychological, physical (sight, hearing), language barriers, and technology-related obstacles [4].

#### Continuity of care

According to the NHS England, “*Personalised care is negotiated between a person and their care provider*,* with empathy*,* in a relationship characterised by trust and respect*,* in which the person feels seen*,* heard and recognised as a person. It works best when the person and their care provider know each other and are prepared*.” [4] Findings also aligned with available guidelines. Patients expressed a preference for teleconsultations with a physician they already know and with whom they have an established relationship. Some results indicated that teleconsultation had a positive effect on patient engagement and access to care, as patients could more easily access teleconsultation than in-person visits. However, patients reported a deterioration in continuity of care, partly due to consulting multiple physicians by teleconsultation, lack of intimacy, and reduced physical and visual contact, which contributed to a perception of reduced physician involvement.

#### Privacy and confidentiality

Privacy results were somewhat misaligned with the guidelines. The use of digital tools was seen as introducing a privacy risk by patients, for instance by enabling physicians to record audio or video. Additionally, the option for patients to conduct consultations from their location increased the risk of being overheard or to be limited in what they could say. Literature occasionally mentioned that patients may be in inappropriate settings during teleconsultations (e.g., with family present, in transit, or in public places), especially when GPs called back at their convenience time. Privacy and confidentiality issues are well discussed in guidelines. These issues are related to devices (e.g., having a private connection), data security, patients and HCPs environment (i.e., having a quiet, safe, and confidential place), or patients and HCPs identities (i.e., confirming information to ensure ID) [4].

## Conclusion

Although guidelines proposed by countries that had adopted teleconsultation generally aligned with patient preferences, some points of divergence remain. These differences are either intrinsic to individuals or organizational in nature. Clearer guidelines on teleconsultation use—regarding its format (phone or video), consultation purposes, the patient-physician relationship, and security protocols—are necessary to prioritize continuity and safety of care while also ensuring and preserving patient engagement in their care pathway. Policies that promote equitable access to teleconsultation for all are also essential as disparities persist among populations, particularly in patients’ interactions with technology. Overall, this review reinforces the need for a hybrid care model that integrates teleconsultation with face-to-face visits, ensuring flexibility to accommodate diverse patient preferences and clinical needs. Policymakers and practitioners must address barriers such as privacy concerns, technological disparities, and diagnostic accuracy while promoting guidelines that prioritize patient-centered care.

## Supplementary Information

Supplementary Material 1.

Supplementary Material 2.

## Data Availability

The registered protocol can be found here: https://www.crd.york.ac.uk/PROSPERO/display_record.php?RecordID=467942. The data that support the findings of this study are available in additional file 3.

## Abbreviations

F2F: Face-to-face consultation
GP: General practitioner
HCP: Healthcare provider
ICT: Information and communication technology
MMAT: Mixed Method Appraisal Tool
PCP: Primary care physician
PRISMA: Preferred Reporting Items for Systematic Reviews and Meta-Analyses
PROSPERO: International Prospective Register of Systematic Reviews
TC: Teleconsultation
WHO: World Health Organization
