# Supplementary material for: Patients’ perspective about synchronous teleconsultation with a general practitioner: a mixed-method systematic literature review
Source: BMC Prim Care. 2025 Aug 20;26:259. doi: 10.1186/s12875-025-02931-w (PMC12366218; doi:10.1186/s12875-025-02931-w)
Supplement: Supplementary file 2 — Supplementary Material 2. [file 12875_2025_2931_MOESM2_ESM.docx]

**Title**: Patients' perspective about synchronous teleconsultation with a general practitioner: A systematic literature review

**Additional Files**

[1. PRISMA 2020 item checklist 2](#_Toc185014802)

[2. Complete search strategy 5](#_Toc185014803)

[3. Complete data extraction 7](#_Toc185014804)

[4. Quality assessment (Mixed Methods Appraisal Tool) 8](#_Toc185014805)

[5. Results - Consultation purpose 10](#_Toc185014806)

[6. Results - Consultation setting 12](#_Toc185014807)

[7. Results - Quality of care 13](#_Toc185014808)

[8. Results - Role of consultation and technology-related 15](#_Toc185014809)

[9. Results - Provider-related 16](#_Toc185014810)

[10. Results - Patient's sociodemographic characteristics and medical condition 17](#_Toc185014811)

[11. Results - Patient's abilities, experience, and attitude towards teleconsultation 20](#_Toc185014812)

[12. Results - Convenience for the patient 22](#_Toc185014813)

[13. Results - Patient-centered care 24](#_Toc185014814)

[14. Results - Institution-related 26](#_Toc185014815)

[15. Number of occurrences by theme or subtheme 28](#_Toc185014816)

[16. Number of occurrences - Consultation purposes only 30](#_Toc185014817)

[17. Number of determinants of teleconsultation use per study 31](#_Toc185014818)

[18. References 32](#_Toc185014819)

Additional file 1 – PRISMA 2020 item checklist

| Section and topic | Item # | Checklist item | Location where item is reported |
| --- | --- | --- | --- |
| Title | | | |
| Title | 1 | Identify the report as a systematic review. | First page |
| Abstract | | | |
| Abstract | 2 | See the PRISMA 2020 for Abstracts checklist (table 2). | First page |
| Introduction | | | |
| Rationale | 3 | Describe the rationale for the review in the context of existing knowledge. | Section 1 (Introduction), §4 |
| Objectives | 4 | Provide an explicit statement of the objective(s) or question(s) the review addresses. | Section 1 (Introduction), §5 |
| Methods | | | |
| Eligibility criteria | 5 | Specify the inclusion and exclusion criteria for the review and how studies were grouped for the syntheses. | Section 2.2 (Selection criteria), §2 |
| Information sources | 6 | Specify all databases, registers, websites, organizations, reference lists and other sources searched or consulted to identify studies. Specify the date when each source was last searched or consulted. | Section 2.1 (Search strategy), §1 |
| Search strategy | 7 | Present the full search strategies for all databases, registers and websites, including any filters and limits used. | Section 2.1 (Search strategy), §1 |
| Selection process | 8 | Specify the methods used to decide whether a study met the inclusion criteria of the review, including how many reviewers screened each record and each report retrieved, whether they worked independently, and if applicable, details of automation tools used in the process. | Section 2.4 (Selection process and data extraction) |
| Data collection process | 9 | Specify the methods used to collect data from reports, including how many reviewers collected data from each report, whether they worked independently, any processes for obtaining or confirming data from study investigators, and if applicable, details of automation tools used in the process. | Section 2.4 (Selection process and data extraction) |
| Data items | 10a | List and define all outcomes for which data were sought. Specify whether all results that were compatible with each outcome domain in each study were sought (e.g. for all measures, time points, analyses), and if not, the methods used to decide which results to collect. | Section 2.4 (Selection process and data extraction), §5 |
|  | 10b | List and define all other variables for which data were sought (e.g. participant and intervention characteristics, funding sources). Describe any assumptions made about any missing or unclear information. | n.a. |
| Study risk of bias assessment | 11 | Specify the methods used to assess risk of bias in the included studies, including details of the tool(s) used, how many reviewers assessed each study and whether they worked independently, and if applicable, details of automation tools used in the process. | Section 2.5 (Quality assessment) |
| Effect measures | 12 | Specify for each outcome the effect measure(s) (e.g. risk ratio, mean difference) used in the synthesis or presentation of results. | Section 2.4 (Selection process and data extraction), §5 |
| Synthesis methods | 13a | Describe the processes used to decide which studies were eligible for each synthesis (e.g. tabulating the study intervention characteristics and comparing against the planned groups for each synthesis (item #5)) | n.a. |
|  | 13b | Describe any methods required to prepare the data for presentation or synthesis, such as handling of missing summary statistics, or data conversions. | Section 2.4 (Selection process and data extraction), §4 |
|  | 13c | Describe any methods used to tabulate or visually display results of individual studies and syntheses. | Section 2.4 (Selection process and data extraction), §4 |
|  | 13d | Describe any methods used to synthesize results and provide a rationale for the choice(s). If meta-analysis was performed, describe the model(s), method(s) to identify the presence and extent of statistical heterogeneity, and software package(s) used. | n.a. |
|  | 13e | Describe any methods used to explore possible causes of heterogeneity among study results (e.g. subgroup analysis, meta-regression). | n.a. |
|  | 13f | Describe any sensitivity analyses conducted to assess robustness of the synthesized results. | n.a. |
| Reporting bias assessment | 14 | Describe any methods used to assess risk of bias due to missing results in a synthesis (arising from reporting biases). | n.a. |
| Certainty assessment | 15 | Describe any methods used to assess certainty (or confidence) in the body of evidence for an outcome. | n.a. |
| Results | | | |
| Study selection | 16a | Describe the results of the search and selection process, from the number of records identified in the search to the number of studies included in the review, ideally using a flow diagram (see fig 1). | Section 3.1 (Studies details) |
|  | 16b | Cite studies that might appear to meet the inclusion criteria, but which were excluded, and explain why they were excluded. | Section 3.1 (Studies details) |
| Study characteristics | 17 | Cite each included study and present its characteristics. | Section 3.1 (Studies details), section 3.1.1 (Studies years and locations), section 3.1.2 (Studies populations), table 2 (Studies details) |
| Risk of bias in studies | 18 | Present assessments of risk of bias for each included study. | n.a. |
| Results of individual studies | 19 | For all outcomes, present, for each study: (a) summary statistics for each group (where appropriate) and (b) an effect estimate and its precision (e.g. confidence/credible interval), ideally using structured tables or plots. | n.a. |
| Results of syntheses | 20a | For each synthesis, briefly summarize the characteristics and risk of bias among contributing studies. | n.a. |
|  | 20b | Present results of all statistical syntheses conducted. If meta-analysis was done, present for each the summary estimate and its precision (e.g. confidence/credible interval) and measures of statistical heterogeneity. If comparing groups, describe the direction of the effect. | n.a. |
|  | 20c | Present results of all investigations of possible causes of heterogeneity among study results. | n.a. |
|  | 20d | Present results of all sensitivity analyses conducted to assess the robustness of the synthesized results. | n.a. |
| Reporting biases | 21 | Present assessments of risk of bias due to missing results (arising from reporting biases) for each synthesis assessed. | n.a. |
| Certainty of evidence | 22 | Present assessments of certainty (or confidence) in the body of evidence for each outcome assessed. | n.a. |
| Discussion | | | |
| Discussion | 23a | Provide a general interpretation of the results in the context of other evidence. | Section 4.1 (Interpretation of the results in the context of existing literature) |
|  | 23b | Discuss any limitations of the evidence included in the review. | Section 4.2 (Strengths and limits of the systematic review) |
|  | 23c | Discuss any limitations of the review processes used. | Section 4.2 (Strengths and limits of the systematic review), §3 |
|  | 23d | Discuss implications of the results for practice, policy, and future research. | Section 4.3 (Policy implications and future research) |
| Other information | | | |
| Registration and protocol | 24a | Provide registration information for the review, including register name and registration number, or state that the review was not registered. | Section 2 (Methods), §3 |
|  | 24b | Indicate where the review protocol can be accessed, or state that a protocol was not prepared. | Section 2 (Methods), §3 |
|  | 24c | Describe and explain any amendments to information provided at registration or in the protocol. | Section 2 (Methods), §3 |
| Support | 25 | Describe sources of financial or non-financial support for the review, and the role of the funders or sponsors in the review. | Section Funding |
| Competing interests | 26 | Declare any competing interests of review authors. | Section Declaration of Interest |
| Availability of data, code, and other materials | 27 | Report which of the following are publicly available and where they can be found: template data collection forms; data extracted from included studies; data used for all analyses; analytic code; any other materials used in the review. | Section Data availability statement |

Additional file 2 – Complete search strategy

***PubMed***

((accept*[Title/Abstract]) OR (adopt*[Title/Abstract]) OR (experience*[Title/Abstract]) OR (participat*[Title/Abstract]) OR (preference*[Title/Abstract]) OR (satisf*[Title/Abstract]) OR (perceive*[Title/Abstract]) OR (perception*[Title/Abstract]) OR (usage[Title/Abstract]) OR (use[Title/Abstract]) OR (user*[Title/Abstract]) OR (utilisation[Title/Abstract]) OR (utilization[Title/Abstract]) OR (utility[Title/Abstract])) AND ((family medicine[Title/Abstract]) OR (general practi*[Title/Abstract]) OR (family practi*[Title/Abstract]) OR (family physician*[Title/Abstract]) OR (family doctor*[Title/Abstract]) OR (general doctor*[Title/Abstract]) OR (primary care[Title/Abstract]) OR (General Practice*[MeSH Terms]) OR (General Practitioners[MeSH Terms]) OR (Physicians, Primary Care[MeSH Terms])) AND (("distance counseling"[Title/Abstract]) OR ("distance counselling"[Title/Abstract]) OR ("tele_consultation*"[Title/Abstract]) OR ("tele_health"[Title/Abstract]) OR ("telehealth*"[Title/Abstract]) OR ("teleconsultation*"[Title/Abstract]) OR ("online medical consultation*"[Title/Abstract]) OR ("remote consultation*"[Title/Abstract]) OR ("tele_medic*"[Title/Abstract]) OR ("telemedic*"[Title/Abstract]) OR ("tele_care"[Title/Abstract]) OR ("telecare"[Title/Abstract]) OR ("video_consultation*"[Title/Abstract]) OR ("virtual consultation*"[Title/Abstract]) OR (Telemedicine*[MeSH Terms]))

***Web of Science***

(TS=(accept*) OR TS=(adopt*) OR TS=(experience*) OR TS=(participat*) OR TS=(preference*) OR TS=(satisf*) OR TS=(perceive*) OR TS=(perception*) OR TS=(usage) OR TS=(use) OR TS=(user*) OR TS=(utilisation) OR TS=(utilization) OR TS=(utility)) AND (TS=(family medicine) OR TS=(general practi*) OR TS=(family practi*) OR TS=(family physician*) OR TS=(family doctor*) OR TS=(general doctor*) OR TS=(primary care)) AND (TS=("distance counseling") OR TS=("distance counselling") OR TS=("tele_health") OR TS=("telehealth") OR TS=("tele_consultation*") OR TS=("teleconsultation*") OR TS=("online medical consultation*") OR TS=("remote consultation*") OR TS=("tele_medic*") OR TS=("telemedic*") OR TS=("tele_care") OR TS=("telecare") OR TS=("video_consultation*") OR TS=("virtual consultation*"))

***Cochrane***

| #1 | (family medicine):ti,ab,kw OR (general practi*):ti,ab,kw OR (family practi*):ti,ab,kw OR (family physician*):ti,ab,kw OR (family doctor*):ti,ab,kw |
| --- | --- |
| #2 | (general doctor*):ti,ab,kw OR (primary care):ti,ab,kw |
| #3 | MeSH descriptor: [General Practice] explode all trees |
| #4 | MeSH descriptor: [General Practitioners] explode all trees |
| #5 | MeSH descriptor: [Physicians, Primary Care] explode all trees |
| #6 | ("distance counseling"):ti,ab,kw OR ("distance counselling"):ti,ab,kw OR ("tele_consultation*"):ti,ab,kw OR ("teleconsultation*"):ti,ab,kw OR ("online medical consultation*"):ti,ab,kw |
| #7 | ("remote consultation*"):ti,ab,kw OR ("tele_medic*"):ti,ab,kw OR ("telemedic*"):ti,ab,kw OR ("tele_care"):ti,ab,kw OR ("telecare"):ti,ab,kw |
| #8 | ("video_consultation*"):ti,ab,kw OR ("virtual consultation*"):ti,ab,kw OR ("tele_health"):ti,ab,kw OR ("telehealth"):ti,ab,kw |
| #9 | MeSH descriptor: [Telemedicine] explode all trees |
| #10 | MeSH descriptor: [Remote Consultation] explode all trees |
| #11 | (accept*):ti,ab,kw OR (adopt*):ti,ab,kw OR (experience*):ti,ab,kw OR (participat*):ti,ab,kw OR (preference*):ti,ab,kw |
| #12 | (satisf*):ti,ab,kw OR (perceive*):ti,ab,kw OR (perception*):ti,ab,kw OR (usage):ti,ab,kw OR (use):ti,ab,kw |
| #13 | (user*):ti,ab,kw OR (utilisation):ti,ab,kw OR (utilization):ti,ab,kw OR (utility):ti,ab,kw |
| #14 | #1 OR #2 OR #3 OR #4 OR #5 |
| #15 | #6 OR #7 OR #8 OR #9 OR #10 |
| #16 | #11 OR #12 OR #13 |
| #17 | #14 AND #15 AND #16 |

***EBSCO***

| S1 | AB family medicine OR AB general practi* OR AB family practi* OR AB family physician* OR AB family doctor* OR AB general doctor* OR AB primary care |
| --- | --- |
| S2 | AB "distance counseling" OR AB "distance counselling" OR AB "tele_consultation*" OR AB "teleconsultation*" OR AB "online medical consultation*" OR AB "remote consultation*" OR AB "tele_medic*" OR AB "telemedic*" OR AB "tele_care" OR AB "telecare" OR AB "video_consultation*" OR AB "virtual consultation*" OR AB "tele_health" OR AB "telehealth" |
| S3 | AB accept* OR AB adopt* OR AB experience* OR AB participat* OR AB preference* OR AB satisf* OR AB perceive* OR AB perception* OR AB usage OR AB use OR AB user* OR AB utilisation OR AB utilization OR AB utility |
| S4 | TI family medicine OR TI general practi* OR TI family practi* OR TI family physician* OR TI family doctor* OR TI general doctor* OR TI primary care |
| S5 | TI "distance counseling" OR TI "distance counselling" OR TI "tele_consultation*" OR TI "teleconsultation*" OR TI "online medical consultation*" OR TI "remote consultation*" OR TI "tele_medic*" OR TI "telemedic*" OR TI "tele_care" OR TI "telecare" OR TI "video_consultation*" OR TI "virtual consultation*" OR TI "tele_health" OR TI "telehealth" |
| S6 | TI accept* OR TI adopt* OR TI experience* OR TI participat* OR TI preference* OR TI satisf* OR TI perceive* OR TI perception* OR TI usage OR TI use OR TI user* OR TI utilisation OR TI utilization OR TI utility |
|  | (S1 AND S2 AND S3) |
|  | (S4 AND S5 AND S6) |
|  | (S1 AND S2 AND S3) OR (S4 AND S5 AND S6) |

Additional file 3 – Complete data extraction

See Excel file.

Additional file 4 – Quality assessment (Mixed Methods Appraisal Tool)

| ID | SCREENING QUESTIONS | | 1. QUALITATIVE STUDIES | | | | | 2. RANDOMIZED CONTROLLED TRIALS | | | | | 3. NON-RANDOMIZED STUDIES | | | | | 4. QUANTITATIVE DESCRIPTIVE STUDIES | | | | | 5. MIXED METHODS STUDIES | | | | |
| --- | --- | --- | --- | --- | --- | --- | --- | --- | --- | --- | --- | --- | --- | --- | --- | --- | --- | --- | --- | --- | --- | --- | --- | --- | --- | --- | --- |
|  | S1 | S2 | 1.1. | 1.2. | 1.3. | 1.4. | 1.5. | 2.1. | 2.2. | 2.3. | 2.4. | 2.5. | 3.1. | 3.2. | 3.3. | 3.4. | 3.5. | 4.1. | 4.2. | 4.3. | 4.4. | 4.5. | 5.1. | 5.2. | 5.3. | 5.4. | 5.5. |
| Abraham, 2022 (1) | Yes | Yes | NA | NA | NA | NA | NA | NA | NA | NA | NA | NA | NA | NA | NA | NA | NA | Can't tell | Can't tell | No | No | No | NA | NA | NA | NA | NA |
| Adams, 2023 (2) | Yes | Yes | Yes | Yes | Yes | Yes | Yes | NA | NA | NA | NA | NA | NA | NA | NA | NA | NA | NA | NA | NA | NA | NA | NA | NA | NA | NA | NA |
| Aghajafari, 2022 (3) | Yes | Yes | Yes | Yes | Yes | Yes | Yes | NA | NA | NA | NA | NA | NA | NA | NA | NA | NA | NA | NA | NA | NA | NA | NA | NA | NA | NA | NA |
| Anderson, 2021 (4) | Yes | Yes | No | No | No | Yes | No | NA | NA | NA | NA | NA | NA | NA | NA | NA | NA | NA | NA | NA | NA | NA | NA | NA | NA | NA | NA |
| Atherton, 2018 (5) | Yes | Yes | Yes | Yes | Yes | No | No | NA | NA | NA | NA | NA | NA | NA | NA | NA | NA | NA | NA | NA | NA | NA | NA | NA | NA | NA | NA |
| Bali, 2007 (6) | Yes | Yes | No | Yes | No | No | No | NA | NA | NA | NA | NA | NA | NA | NA | NA | NA | NA | NA | NA | NA | NA | NA | NA | NA | NA | NA |
| Ball, 2018 (7) | Yes | Yes | Can't tell | Yes | Yes | Yes | Yes | NA | NA | NA | NA | NA | NA | NA | NA | NA | NA | NA | NA | NA | NA | NA | NA | NA | NA | NA | NA |
| Bhatia, 2022 (8) | Yes | Yes | Yes | Yes | Yes | Yes | Yes | NA | NA | NA | NA | NA | NA | NA | NA | NA | NA | Yes | Yes | Yes | Yes | Yes | No | Yes | Yes | Yes | Yes |
| Binder-Olibrowska, 2022 (9) | Yes | Yes | No | Yes | Yes | Yes | No | NA | NA | NA | NA | NA | NA | NA | NA | NA | NA | Yes | Yes | Yes | Can't tell | Yes | No | Yes | Yes | No | No |
| Bittleston, 2022 (10) | Yes | Yes | Can't tell | Yes | Yes | Yes | No | NA | NA | NA | NA | NA | NA | NA | NA | NA | NA | NA | NA | NA | NA | NA | NA | NA | NA | NA | NA |
| Brown, 1995 (11) | Yes | Yes | NA | NA | NA | NA | NA | NA | NA | NA | NA | NA | No | No | Yes | No | Can't tell | NA | NA | NA | NA | NA | NA | NA | NA | NA | NA |
| Buchanan, 2021 (12) | Yes | Yes | NA | NA | NA | NA | NA | NA | NA | NA | NA | NA | NA | NA | NA | NA | NA | Yes | Yes | Yes | Yes | Yes | NA | NA | NA | NA | NA |
| Burton, 2022 (13) | Yes | Yes | Can't tell | Yes | Yes | Yes | Yes | NA | NA | NA | NA | NA | NA | NA | NA | NA | NA | NA | NA | NA | NA | NA | NA | NA | NA | NA | NA |
| Chen, 2022 (14) | Yes | Yes | NA | NA | NA | NA | NA | NA | NA | NA | NA | NA | Yes | No | Can't tell | Yes | Can't tell | NA | NA | NA | NA | NA | NA | NA | NA | NA | NA |
| Chudner, 2019a (15) | Yes | Yes | Yes | Yes | Yes | Yes | Yes | NA | NA | NA | NA | NA | NA | NA | NA | NA | NA | NA | NA | NA | NA | NA | NA | NA | NA | NA | NA |
| Chudner, 2019b (16) | Yes | Yes | NA | NA | NA | NA | NA | NA | NA | NA | NA | NA | NA | NA | NA | NA | NA | Yes | Yes | Yes | Yes | Yes | NA | NA | NA | NA | NA |
| Ciecko, 2023 (17) | Yes | Yes | NA | NA | NA | NA | NA | NA | NA | NA | NA | NA | NA | NA | NA | NA | NA | No | Can't tell | No | Can't tell | No | NA | NA | NA | NA | NA |
| Curtis, 2021 (18) | Yes | Yes | Can't tell | Yes | Yes | Yes | Yes | NA | NA | NA | NA | NA | NA | NA | NA | NA | NA | Yes | Yes | Yes | Yes | Yes | No | Yes | Yes | Yes | No |
| Devillers, 2023 (19) | Yes | Yes | NA | NA | NA | NA | NA | NA | NA | NA | NA | NA | NA | NA | NA | NA | NA | Yes | Can't tell | No | Can't tell | No | NA | NA | NA | NA | NA |
| Dixon, 2008 (20) | Yes | Yes | NA | NA | NA | NA | NA | Yes | No | Can't tell | No | Yes | NA | NA | NA | NA | NA | NA | NA | NA | NA | NA | NA | NA | NA | NA | NA |
| Dixon, 2009 (21) | Yes | Yes | NA | NA | NA | NA | NA | No | No | Can't tell | No | Yes | NA | NA | NA | NA | NA | NA | NA | NA | NA | NA | NA | NA | NA | NA | NA |
| Donaghy, 2019 (22) | Yes | Yes | Yes | Yes | Yes | Yes | Yes | NA | NA | NA | NA | NA | NA | NA | NA | NA | NA | NA | NA | NA | NA | NA | NA | NA | NA | NA | NA |
| Donaghy, 2023 (23) | Yes | Yes | No | Yes | Yes | Yes | Yes | NA | NA | NA | NA | NA | NA | NA | NA | NA | NA | Yes | Can't tell | Can't tell | Yes | No | Yes | Yes | Can't tell | Can't tell | No |
| Duncan, 2021 (24) | Yes | Yes | Yes | Yes | Yes | Yes | No | NA | NA | NA | NA | NA | NA | NA | NA | NA | NA | NA | NA | NA | NA | NA | NA | NA | NA | NA | NA |
| Esber, 2023 (25) | Yes | Yes | NA | NA | NA | NA | NA | NA | NA | NA | NA | NA | NA | NA | NA | NA | NA | Yes | Yes | Yes | Yes | Yes | NA | NA | NA | NA | NA |
| Garrett, 2022 (26) | Yes | Yes | Yes | Yes | Yes | Yes | Yes | NA | NA | NA | NA | NA | NA | NA | NA | NA | NA | NA | NA | NA | NA | NA | NA | NA | NA | NA | NA |
| Greenhalgh, 2022 (27) | Yes | Yes | Yes | Yes | Yes | Yes | Yes | NA | NA | NA | NA | NA | NA | NA | NA | NA | NA | NA | NA | NA | NA | NA | NA | NA | NA | NA | NA |
| Han, 2022 (28) | Yes | Yes | Yes | Yes | Yes | Yes | Yes | NA | NA | NA | NA | NA | NA | NA | NA | NA | NA | NA | NA | NA | NA | NA | NA | NA | NA | NA | NA |
| Assing Hvidt, 2022 (29) | Yes | Yes | Yes | Yes | Yes | Yes | Yes | NA | NA | NA | NA | NA | NA | NA | NA | NA | NA | NA | NA | NA | NA | NA | NA | NA | NA | NA | NA |
| Javanparast, 2021a (30) | Yes | Yes | Yes | Yes | Yes | Yes | Yes | NA | NA | NA | NA | NA | NA | NA | NA | NA | NA | NA | NA | NA | NA | NA | NA | NA | NA | NA | NA |
| Javanparast, 2021b (31) | Yes | Yes | Yes | Yes | Yes | Yes | Yes | NA | NA | NA | NA | NA | NA | NA | NA | NA | NA | NA | NA | NA | NA | NA | NA | NA | NA | NA | NA |
| Kludacz-Alessandri, 2021 (32) | Yes | Yes | NA | NA | NA | NA | NA | NA | NA | NA | NA | NA | NA | NA | NA | NA | NA | Yes | Can't tell | Yes | Yes | Yes | NA | NA | NA | NA | NA |
| Kowalski, 2018 (33) | Yes | Yes | NA | NA | NA | NA | NA | NA | NA | NA | NA | NA | NA | NA | NA | NA | NA | Yes | No | Yes | Can't tell | No | NA | NA | NA | NA | NA |
| Leng, 2016 (34) | Yes | Yes | Yes | Yes | Yes | No | No | NA | NA | NA | NA | NA | NA | NA | NA | NA | NA | Yes | Yes | No | Yes | No | Yes | Yes | No | Can't tell | No |
| Mangalji, 2022 (35) | Yes | Yes | NA | NA | NA | NA | NA | NA | NA | NA | NA | NA | NA | NA | NA | NA | NA | Yes | Yes | Can't tell | Can't tell | No | NA | NA | NA | NA | NA |
| Manski-Nankervis, 2022 (36) | Yes | Yes | NA | NA | NA | NA | NA | NA | NA | NA | NA | NA | NA | NA | NA | NA | NA | Yes | Yes | Yes | No | Yes | NA | NA | NA | NA | NA |
| Mathew, 2021 (37) | Yes | Yes | NA | NA | NA | NA | NA | NA | NA | NA | NA | NA | NA | NA | NA | NA | NA | Yes | Yes | Yes | Can't tell | Yes | NA | NA | NA | NA | NA |
| McKinstry, 2009 (38) | Yes | Yes | Yes | Yes | Yes | Yes | Yes | NA | NA | NA | NA | NA | NA | NA | NA | NA | NA | NA | NA | NA | NA | NA | NA | NA | NA | NA | NA |
| McKinstry, 2010 (39) | Yes | Yes | NA | NA | NA | NA | NA | NA | NA | NA | NA | NA | NA | NA | NA | NA | NA | Yes | No | Yes | Yes | Yes | NA | NA | NA | NA | NA |
| Mohan, 2022 (40) | Yes | Yes | NA | NA | NA | NA | NA | NA | NA | NA | NA | NA | NA | NA | NA | NA | NA | Yes | Yes | Yes | No | No | NA | NA | NA | NA | NA |
| Payne, 2001 (41) | Yes | Yes | Yes | Yes | Yes | Yes | No | NA | NA | NA | NA | NA | NA | NA | NA | NA | NA | NA | NA | NA | NA | NA | NA | NA | NA | NA | NA |
| Poitras, 2022 (42) | Yes | Yes | Yes | Yes | Yes | Yes | Yes | NA | NA | NA | NA | NA | NA | NA | NA | NA | NA | NA | NA | NA | NA | NA | NA | NA | NA | NA | NA |
| Powell, 2017 (43) | Yes | Yes | Yes | Yes | Yes | Yes | Yes | NA | NA | NA | NA | NA | NA | NA | NA | NA | NA | NA | NA | NA | NA | NA | NA | NA | NA | NA | NA |
| Reed, 2020 (44) | Yes | Yes | NA | NA | NA | NA | NA | NA | NA | NA | NA | NA | NA | NA | NA | NA | NA | Yes | Yes | No | Yes | Yes | NA | NA | NA | NA | NA |
| Rose, 2021 (45) | Yes | Yes | Yes | Yes | Yes | Yes | Yes | NA | NA | NA | NA | NA | NA | NA | NA | NA | NA | Yes | Yes | Yes | Can't tell | Yes | Yes | Yes | Yes | Can't tell | Can't tell |
| von Weinrich, 2022 (46) | Yes | Yes | NA | NA | NA | NA | NA | NA | NA | NA | NA | NA | NA | NA | NA | NA | NA | Yes | Yes | Yes | No | Yes | NA | NA | NA | NA | NA |

Additional file 5 – Results - Consultation purpose

| Author first name, year | Consultation purpose | | | | | | | | | | | | | | | | | | | |
| --- | --- | --- | --- | --- | --- | --- | --- | --- | --- | --- | --- | --- | --- | --- | --- | --- | --- | --- | --- | --- |
|  | Health issues | | | | | | | | | | Administrative and documentation | | | Assessment and guidance | | | | | | Other (non-specified) consultation purpose |
|  | Physical and mental functioning | | | Disease time course | | Child health issues | Complexity and severity of the health issue | Emergency health issues | Sensitive or personal issues | Others (Health issues) | Prescription renewal/refill | Referring (e.g., specialist, lab) | Obtaining certificate (e.g., sick leave) | General advice | Diagnosis | Need a visual/physical examination | Discussing treatment | Request tests and discussing test results | Others (Assessment and guidance) |  |
|  | Mobility issues | Mental health issues | Dermatological conditions (e.g., rashes) | Follow-up/Routine consultation | New health issues |  |  |  |  |  |  |  |  |  |  |  |  |  |  |  |
| Adams, 2023 (2) | 0 | 1 | 0 | 0 | 0 | 0 | 1 | 0 | 0 | 0 | 1 | 0 | 0 | 0 | 0 | 1 | 0 | 0 | 0 | 0 |
| Aghajafari, 2022 (3) | 0 | 0 | 0 | 0 | 0 | 0 | 0 | 0 | 1 | 0 | 0 | 0 | 0 | 0 | 0 | 0 | 0 | 0 | 0 | 0 |
| Assing Hvidt, 2022 (29) | 0 | 1 | 1 | 1 | 0 | 0 | 1 | 0 | 0 | 0 | 0 | 0 | 0 | 0 | 0 | 1 | 0 | 1 | 0 | 0 |
| Atherton, 2018 (5) | 0 | 0 | 0 | 0 | 1 | 0 | 0 | 0 | 0 | 1 | 0 | 0 | 0 | 1 | 0 | 0 | 0 | 0 | 0 | 0 |
| Bali, 2007 (6) | 0 | 1 | 1 | 1 | 0 | 0 | 0 | 0 | 0 | 1 | 0 | 1 | 0 | 1 | 0 | 0 | 1 | 0 | 1 | 0 |
| Ball, 2018 (7) | 0 | 0 | 0 | 0 | 0 | 0 | 0 | 1 | 0 | 0 | 0 | 0 | 0 | 0 | 0 | 0 | 0 | 0 | 0 | 0 |
| Bhatia, 2022 (8) | 0 | 0 | 0 | 1 | 0 | 0 | 1 | 0 | 0 | 0 | 0 | 0 | 0 | 0 | 1 | 0 | 0 | 0 | 0 | 0 |
| Binder-Olibrowska, 2022 (9) | 0 | 0 | 0 | 0 | 0 | 0 | 0 | 1 | 0 | 0 | 0 | 0 | 1 | 0 | 0 | 0 | 1 | 0 | 0 | 0 |
| Bittleston, 2022 (10) | 0 | 0 | 0 | 0 | 0 | 0 | 1 | 0 | 0 | 0 | 1 | 0 | 0 | 0 | 0 | 0 | 0 | 0 | 0 | 0 |
| Ciecko, 2023 (17) | 0 | 0 | 0 | 0 | 0 | 0 | 0 | 0 | 0 | 0 | 1 | 0 | 0 | 0 | 0 | 0 | 0 | 0 | 0 | 0 |
| Curtis, 2021 (18) | 0 | 1 | 0 | 0 | 0 | 0 | 1 | 0 | 1 | 0 | 0 | 0 | 0 | 0 | 0 | 1 | 1 | 0 | 0 | 1 |
| Donaghy, 2019 (22) | 0 | 1 | 0 | 1 | 0 | 0 | 0 | 0 | 1 | 0 | 0 | 0 | 0 | 0 | 0 | 0 | 1 | 1 | 0 | 0 |
| Garrett, 2022 (26) | 0 | 1 | 0 | 0 | 0 | 0 | 1 | 0 | 1 | 0 | 1 | 0 | 0 | 0 | 1 | 0 | 0 | 1 | 0 | 0 |
| Greenhalgh, 2022 (27) | 0 | 0 | 0 | 0 | 0 | 0 | 0 | 0 | 0 | 0 | 0 | 0 | 0 | 0 | 0 | 0 | 0 | 0 | 0 | 1 |
| Han, 2022 (28) | 0 | 1 | 1 | 0 | 0 | 0 | 1 | 0 | 1 | 0 | 0 | 0 | 0 | 0 | 0 | 0 | 0 | 0 | 0 | 0 |
| Javanparast, 2021a (30) | 0 | 1 | 0 | 0 | 0 | 0 | 1 | 0 | 0 | 0 | 1 | 0 | 0 | 0 | 0 | 0 | 0 | 1 | 0 | 0 |
| Javanparast, 2021b (31) | 0 | 0 | 0 | 1 | 0 | 0 | 0 | 0 | 0 | 0 | 1 | 0 | 0 | 0 | 0 | 0 | 0 | 1 | 0 | 0 |
| Kludacz-Alessandri, 2021 (32) | 0 | 0 | 0 | 0 | 0 | 0 | 0 | 0 | 0 | 0 | 0 | 0 | 0 | 0 | 0 | 0 | 0 | 0 | 0 | 1 |
| Leng, 2016 (34) | 1 | 1 | 1 | 1 | 0 | 1 | 1 | 1 | 1 | 0 | 1 | 0 | 1 | 1 | 0 | 1 | 0 | 1 | 0 | 1 |
| McKinstry, 2009 (38) | 0 | 0 | 1 | 1 | 0 | 1 | 1 | 0 | 0 | 1 | 0 | 0 | 0 | 0 | 0 | 1 | 0 | 1 | 0 | 0 |
| McKinstry, 2010 | 0 | 0 | 0 | 1 | 1 | 0 | 0 | 0 | 0 | 0 | 0 | 0 | 0 | 0 | 0 | 0 | 0 | 0 | 0 | 0 |
| Mohan, 2022 (40) | 0 | 0 | 0 | 0 | 0 | 0 | 1 | 0 | 0 | 0 | 1 | 0 | 0 | 0 | 0 | 0 | 0 | 1 | 0 | 0 |
| Poitras, 2022 (42) | 0 | 1 | 0 | 1 | 0 | 0 | 0 | 0 | 1 | 0 | 1 | 0 | 0 | 0 | 0 | 0 | 0 | 1 | 0 | 0 |
| Powell, 2017 (43) | 0 | 0 | 0 | 0 | 0 | 0 | 0 | 0 | 0 | 0 | 0 | 0 | 0 | 0 | 0 | 1 | 0 | 0 | 0 | 1 |

Note: the cell takes the value 1 if the article deals with the teleconsultation determinant of the column.

Additional file 6 – Results - Consultation setting

| Author first name, year | Consultation setting | | | | |
| --- | --- | --- | --- | --- | --- |
|  | Possibility to send documents (e.g., test results, photos, videos) | Concerns about missing appointment due to waiting room setting | Triaging | Ease of appointment booking | Peer support |
| Adams, 2023 (2) | 0 | 1 | 0 | 1 | 0 |
| Aghajafari, 2022 (3) | 0 | 0 | 0 | 0 | 1 |
| Anderson, 2021 (4) | 0 | 1 | 0 | 0 | 0 |
| Assing Hvidt, 2022 (29) | 0 | 1 | 0 | 0 | 0 |
| Ball, 2018 (7) | 0 | 0 | 0 | 1 | 0 |
| Bhatia, 2022 (8) | 1 | 1 | 0 | 0 | 1 |
| Binder-Olibrowska, 2022 (9) | 0 | 0 | 0 | 1 | 0 |
| Bittleston, 2022 (10) | 0 | 0 | 0 | 1 | 0 |
| Burton, 2022 (13) | 0 | 0 | 0 | 1 | 1 |
| Donaghy, 2019 (22) | 0 | 1 | 0 | 0 | 0 |
| Donaghy, 2023 (23) | 0 | 0 | 0 | 0 | 1 |
| Esber, 2023 (25) | 0 | 0 | 0 | 0 | 1 |
| Garrett, 2022 (26) | 0 | 0 | 1 | 0 | 0 |
| Leng, 2016 (34) | 0 | 0 | 1 | 0 | 0 |
| Powell, 2017 (43) | 0 | 0 | 0 | 0 | 1 |
| Reed, 2020 (44) | 0 | 0 | 0 | 0 | 1 |
| Rose, 2021 (45) | 0 | 0 | 0 | 1 | 0 |
| von Weinrich, 2022 (46) | 0 | 0 | 0 | 1 | 0 |

Note: the cell takes the value 1 if the article deals with the teleconsultation determinant of the column.

Additional file 7 – Results - Quality of care

| Author first name, year | Quality of care | | | | | | | | |
| --- | --- | --- | --- | --- | --- | --- | --- | --- | --- |
|  | Teleconsultation undermines the accuracy/reliability | | | | | Duration of consultation | Teleconsultation safety | | Others (Quality of care) |
|  | Lack of/difficulties in physical examinations | Risk of misunderstanding/misdiagnosis/error | Lack of verbal, non-verbal, and behavioral cues | Lack of intimacy | Other (Accuracy/reliability of teleconsultation) |  | General safety | Reduced risk of infection |  |
| Adams, 2023 (2) | 1 | 1 | 1 | 0 | 0 | 0 | 0 | 0 | 0 |
| Aghajafari, 2022 (3) | 0 | 0 | 0 | 0 | 1 | 1 | 0 | 0 | 0 |
| Anderson, 2021 (4) | 0 | 1 | 0 | 0 | 1 | 0 | 0 | 0 | 0 |
| Assing Hvidt, 2022 (29) | 0 | 0 | 1 | 0 | 0 | 1 | 0 | 0 | 0 |
| Atherton, 2018 (5) | 0 | 0 | 0 | 0 | 0 | 0 | 0 | 0 | 1 |
| Bali, 2007 (6) | 0 | 0 | 0 | 0 | 0 | 1 | 0 | 0 | 1 |
| Ball, 2018 (7) | 0 | 1 | 0 | 0 | 0 | 0 | 1 | 0 | 0 |
| Bhatia, 2022 (8) | 1 | 1 | 1 | 1 | 0 | 1 | 0 | 1 | 1 |
| Binder-Olibrowska, 2022 (9) | 1 | 1 | 0 | 0 | 0 | 1 | 0 | 0 | 1 |
| Bittleston, 2022 (10) | 0 | 0 | 1 | 0 | 0 | 0 | 0 | 0 | 0 |
| Ciecko, 2023 (17) | 0 | 0 | 0 | 0 | 0 | 0 | 0 | 0 | 1 |
| Curtis, 2021 (18) | 0 | 0 | 1 | 0 | 0 | 1 | 0 | 0 | 0 |
| Dixon, 2008 (20) | 0 | 0 | 0 | 0 | 0 | 1 | 0 | 0 | 0 |
| Dixon, 2009 (21) | 0 | 0 | 0 | 0 | 0 | 1 | 0 | 0 | 0 |
| Donaghy, 2023 (23) | 0 | 0 | 0 | 0 | 0 | 1 | 0 | 0 | 0 |
| Esber, 2023 (25) | 0 | 0 | 0 | 0 | 0 | 0 | 0 | 0 | 1 |
| Garrett, 2022 (26) | 1 | 1 | 0 | 0 | 0 | 1 | 0 | 0 | 0 |
| Greenhalgh, 2022 (27) | 1 | 0 | 0 | 0 | 0 | 0 | 0 | 0 | 0 |
| Han, 2022 (28) | 1 | 0 | 1 | 0 | 0 | 0 | 0 | 0 | 0 |
| Javanparast, 2021a (30) | 0 | 0 | 1 | 0 | 0 | 1 | 0 | 0 | 0 |
| Leng, 2016 (34) | 0 | 1 | 0 | 1 | 0 | 0 | 0 | 0 | 0 |
| McKinstry, 2009 (38) | 1 | 1 | 1 | 0 | 0 | 1 | 0 | 0 | 0 |
| McKinstry, 2010 (39) | 0 | 0 | 0 | 0 | 0 | 1 | 0 | 0 | 0 |
| Mohan, 2022 (40) | 1 | 0 | 0 | 0 | 0 | 1 | 0 | 0 | 0 |
| Payne, 2001 (41) | 1 | 1 | 0 | 0 | 0 | 0 | 0 | 0 | 0 |
| Poitras, 2022 (42) | 1 | 0 | 0 | 0 | 0 | 0 | 0 | 0 | 0 |
| Powell, 2017 (43) | 0 | 0 | 0 | 1 | 0 | 0 | 0 | 0 | 0 |

Note: the cell takes the value 1 if the article deals with the teleconsultation determinant of the column.

Additional file 8 – Results - Role of consultation and technology-related

| Author first name, year | Role of consultation by phone or video as a modality of consultation | | TC technology related | | |
| --- | --- | --- | --- | --- | --- |
|  | Complementarity/Substitutability between modalities of consultation | Similarities of teleconsultation with face-to-face consultation | Privacy, confidentiality, and security issues | Technical issues | Ability of patient to set-up teleconsultation |
| Adams, 2023 (2) | 0 | 0 | 1 | 0 | 1 |
| Assing Hvidt, 2022 (29) | 1 | 0 | 0 | 1 | 0 |
| Ball, 2018 (7) | 1 | 0 | 1 | 0 | 1 |
| Bhatia, 2022 (8) | 1 | 0 | 0 | 1 | 1 |
| Binder-Olibrowska, 2022 (9) | 1 | 0 | 0 | 1 | 0 |
| Bittleston, 2022 (10) | 0 | 0 | 1 | 1 | 0 |
| Brown, 1995 (11) | 0 | 0 | 0 | 0 | 1 |
| Buchanan, 2021 (12) | 0 | 1 | 0 | 0 | 0 |
| Devillers, 2023 (19) | 1 | 0 | 0 | 1 | 0 |
| Donaghy, 2019 (22) | 0 | 0 | 1 | 1 | 0 |
| Duncan, 2021 (24) | 1 | 0 | 1 | 0 | 1 |
| Garrett, 2022 (26) | 0 | 0 | 1 | 0 | 0 |
| Han, 2022 (28) | 0 | 0 | 1 | 1 | 0 |
| Javanparast, 2021a (30) | 1 | 0 | 0 | 0 | 0 |
| Javanparast, 2021b (31) | 1 | 0 | 0 | 0 | 0 |
| Leng, 2016 (34) | 0 | 0 | 1 | 0 | 0 |
| McKinstry, 2009 (38) | 0 | 0 | 0 | 1 | 0 |
| Payne, 2001 (41) | 1 | 0 | 0 | 0 | 0 |
| Poitras, 2022 (42) | 0 | 1 | 0 | 0 | 1 |
| Powell, 2017 (43) | 1 | 0 | 1 | 1 | 0 |
| Rose, 2021 (45) | 1 | 0 | 0 | 1 | 0 |

Note: the cell takes the value 1 if the article deals with the teleconsultation determinant of the column.

Additional file 9 – Results - Provider-related

| Author first name, year | Provider related | | |
| --- | --- | --- | --- |
|  | Provider's reputation | Provider's ability to communicate | General provider's abilities and knowledge |
| Buchanan, 2021 (12) | 1 | 0 | 0 |
| Payne, 2001 (41) | 0 | 1 | 1 |
| Poitras, 2022 (42) | 0 | 1 | 1 |
| Rose, 2021 (45) | 0 | 1 | 1 |

Note: the cell takes the value 1 if the article deals with the teleconsultation determinant of the column.

Additional file 10 – Results - Patient's sociodemographic characteristics and medical condition

| Author first name, year | Patient's sociodemographic characteristics | | | | | | | | | | | Patient's medical condition | | | | | | | |
| --- | --- | --- | --- | --- | --- | --- | --- | --- | --- | --- | --- | --- | --- | --- | --- | --- | --- | --- | --- |
|  | Patient's socioeconomic profile | | | | | Patient's demographic profile | | | | | Other (Patient's sociodemographic and demographic characteristics) | Diseases of the senses | | Chronic health issue | | Mental health/cognitive problems | Restricted mobility | Disabled | Others (Patient's medical condition) |
|  | Occupation status | Education level | Income | Living remotely | Being uninsured | Gender (female) | Age | Family/Having children | Ethnicity | Language |  | Hearing problems | Visual problems | Regular medication | Chronic condition |  |  |  |  |
| Adams, 2023 (2) | 0 | 0 | 0 | 0 | 0 | 0 | 0 | 0 | 0 | 1 | 0 | 1 | 0 | 0 | 0 | 1 | 0 | 0 | 0 |
| Aghajafari, 2022 (3) | 0 | 0 | 0 | 0 | 0 | 0 | 1 | 0 | 0 | 0 | 0 | 0 | 0 | 0 | 0 | 0 | 0 | 0 | 1 |
| Anderson, 2021 (4) | 0 | 0 | 0 | 0 | 0 | 0 | 1 | 0 | 0 | 0 | 0 | 0 | 0 | 0 | 0 | 0 | 0 | 0 | 0 |
| Assing Hvidt, 2022 (29) | 1 | 0 | 0 | 0 | 0 | 0 | 1 | 1 | 0 | 0 | 0 | 0 | 1 | 0 | 0 | 0 | 0 | 0 | 0 |
| Atherton, 2018 (5) | 0 | 0 | 0 | 0 | 0 | 0 | 1 | 0 | 0 | 0 | 0 | 0 | 0 | 0 | 0 | 0 | 0 | 0 | 0 |
| Bali, 2007 (6) | 0 | 0 | 1 | 1 | 0 | 1 | 1 | 0 | 0 | 0 | 0 | 0 | 0 | 0 | 0 | 0 | 0 | 0 | 1 |
| Ball, 2018 (7) | 1 | 0 | 0 | 0 | 0 | 0 | 1 | 1 | 0 | 0 | 1 | 0 | 0 | 0 | 1 | 1 | 1 | 1 | 0 |
| Bhatia, 2022 (8) | 0 | 0 | 0 | 0 | 0 | 0 | 0 | 0 | 1 | 0 | 0 | 0 | 0 | 0 | 0 | 0 | 1 | 0 | 1 |
| Binder-Olibrowska, 2022 (9) | 0 | 1 | 0 | 1 | 0 | 1 | 1 | 1 | 0 | 0 | 0 | 0 | 1 | 0 | 1 | 0 | 0 | 0 | 0 |
| Brown, 1995 (11) | 0 | 0 | 0 | 0 | 0 | 0 | 0 | 1 | 0 | 0 | 0 | 0 | 0 | 0 | 0 | 0 | 0 | 0 | 1 |
| Burton, 2022 (13) | 0 | 0 | 0 | 0 | 0 | 0 | 0 | 0 | 0 | 0 | 0 | 1 | 0 | 0 | 0 | 0 | 0 | 0 | 0 |
| Chen, 2022 (14) | 0 | 0 | 0 | 0 | 1 | 1 | 1 | 0 | 1 | 1 | 0 | 0 | 0 | 0 | 1 | 0 | 0 | 0 | 0 |
| Chudner, 2019b (16) | 0 | 0 | 0 | 0 | 0 | 0 | 1 | 0 | 0 | 0 | 0 | 0 | 0 | 0 | 0 | 0 | 0 | 0 | 0 |
| Curtis, 2021 (18) | 0 | 0 | 0 | 0 | 0 | 1 | 1 | 0 | 0 | 0 | 0 | 1 | 0 | 0 | 0 | 0 | 0 | 0 | 0 |
| Devillers, 2023 (19) | 0 | 0 | 0 | 0 | 0 | 1 | 0 | 0 | 0 | 0 | 0 | 0 | 0 | 0 | 0 | 0 | 0 | 0 | 0 |
| Donaghy, 2019 (22) | 1 | 0 | 0 | 0 | 0 | 0 | 0 | 1 | 0 | 0 | 0 | 0 | 0 | 0 | 0 | 0 | 0 | 0 | 0 |
| Donaghy, 2023 (23) | 0 | 0 | 0 | 0 | 0 | 0 | 0 | 0 | 0 | 0 | 0 | 1 | 0 | 0 | 0 | 0 | 0 | 0 | 0 |
| Duncan, 2021 (24) | 0 | 0 | 0 | 0 | 0 | 0 | 0 | 0 | 0 | 0 | 0 | 1 | 0 | 0 | 0 | 1 | 0 | 0 | 0 |
| Esber, 2023 (25) | 1 | 0 | 0 | 0 | 0 | 0 | 0 | 0 | 0 | 0 | 0 | 0 | 0 | 1 | 0 | 1 | 0 | 0 | 0 |
| Garrett, 2022 (26) | 0 | 0 | 0 | 0 | 0 | 0 | 1 | 0 | 0 | 1 | 0 | 1 | 0 | 0 | 0 | 0 | 0 | 1 | 1 |
| Greenhalgh, 2022 (27) | 0 | 0 | 0 | 0 | 0 | 0 | 0 | 0 | 0 | 0 | 1 | 0 | 0 | 0 | 0 | 1 | 0 | 0 | 1 |
| Han, 2022 (28) | 0 | 0 | 0 | 0 | 0 | 1 | 1 | 0 | 0 | 0 | 0 | 0 | 0 | 0 | 0 | 1 | 0 | 0 | 1 |
| Javanparast, 2021a (30) | 0 | 0 | 0 | 0 | 0 | 0 | 1 | 0 | 0 | 0 | 0 | 0 | 0 | 0 | 0 | 0 | 0 | 0 | 0 |
| Kludacz-Alessandri, 2021 (32) | 0 | 1 | 0 | 0 | 0 | 0 | 1 | 0 | 0 | 0 | 0 | 0 | 0 | 0 | 0 | 0 | 0 | 0 | 0 |
| Kowalski, 2018 (33) | 0 | 0 | 0 | 0 | 0 | 0 | 1 | 0 | 0 | 0 | 0 | 0 | 0 | 0 | 0 | 0 | 0 | 0 | 0 |
| Leng, 2016 (34) | 1 | 0 | 0 | 1 | 0 | 0 | 1 | 1 | 0 | 1 | 1 | 0 | 0 | 0 | 0 | 0 | 1 | 1 | 0 |
| Manski-Nankervis, 2022 (36) | 0 | 1 | 1 | 1 | 0 | 0 | 0 | 0 | 0 | 0 | 0 | 0 | 0 | 0 | 0 | 0 | 0 | 0 | 0 |
| Mathew, 2021 (37) | 0 | 0 | 0 | 0 | 0 | 0 | 0 | 0 | 0 | 0 | 1 | 0 | 0 | 0 | 0 | 0 | 0 | 0 | 0 |
| McKinstry, 2009 (38) | 1 | 0 | 0 | 1 | 0 | 0 | 0 | 1 | 0 | 0 | 0 | 0 | 0 | 0 | 0 | 0 | 1 | 0 | 0 |
| Mohan, 2022 (40) | 0 | 0 | 0 | 0 | 0 | 0 | 1 | 0 | 0 | 0 | 0 | 0 | 0 | 0 | 0 | 0 | 0 | 0 | 0 |
| Poitras, 2022 (42) | 0 | 0 | 0 | 0 | 0 | 0 | 0 | 1 | 0 | 0 | 0 | 0 | 1 | 0 | 0 | 0 | 0 | 0 | 0 |
| Powell, 2017 (43) | 0 | 0 | 0 | 0 | 0 | 0 | 0 | 1 | 0 | 0 | 0 | 0 | 0 | 0 | 0 | 0 | 0 | 1 | 0 |
| Reed, 2020 (44) | 0 | 0 | 0 | 0 | 0 | 1 | 1 | 0 | 1 | 1 | 1 | 0 | 0 | 0 | 0 | 0 | 0 | 0 | 0 |
| Rose, 2021 (45) | 1 | 0 | 0 | 0 | 0 | 0 | 0 | 1 | 0 | 0 | 0 | 0 | 0 | 0 | 0 | 0 | 0 | 0 | 0 |

Note: the cell takes the value 1 if the article deals with the teleconsultation determinant of the column.

Additional file 11 – Results - Patient's abilities, experience, and attitude towards teleconsultation

| Author first name, year | Patient's abilities, experience, and attitudes towards teleconsultation | | | | | | | |
| --- | --- | --- | --- | --- | --- | --- | --- | --- |
|  | Patient's ability to communicate | Patient's ability to communicate with technology | Patient's ability to use technology | General patient's abilities and knowledge | Patient's attitude towards teleconsultation | | | |
|  |  |  |  |  | Patient's prior preferences for video-consultation over phone-consultation | Patient's prior preferences for face-to-face consultation over teleconsultation | Patient's experience with teleconsultation | General attitudes towards teleconsultation |
| Abraham, 2022 (1) | 0 | 0 | 1 | 0 | 0 | 0 | 0 | 0 |
| Adams, 2023 (2) | 0 | 1 | 0 | 0 | 0 | 0 | 0 | 0 |
| Aghajafari, 2022 (3) | 0 | 1 | 0 | 0 | 1 | 0 | 0 | 0 |
| Anderson, 2021 (4) | 1 | 0 | 0 | 0 | 1 | 0 | 1 | 0 |
| Assing Hvidt, 2022 (29) | 0 | 0 | 0 | 0 | 1 | 0 | 0 | 0 |
| Atherton, 2018 (5) | 0 | 0 | 0 | 0 | 0 | 1 | 0 | 0 |
| Ball, 2018 (7) | 1 | 0 | 0 | 0 | 0 | 0 | 0 | 0 |
| Bhatia, 2022 (8) | 0 | 0 | 1 | 1 | 1 | 0 | 0 | 0 |
| Binder-Olibrowska, 2022 (9) | 0 | 0 | 1 | 0 | 0 | 0 | 0 | 0 |
| Brown, 1995 (11) | 0 | 1 | 0 | 0 | 0 | 0 | 1 | 0 |
| Buchanan, 2021 (12) | 0 | 0 | 0 | 0 | 0 | 1 | 0 | 0 |
| Burton, 2022 (13) | 0 | 0 | 0 | 1 | 0 | 0 | 1 | 0 |
| Chen, 2022 (14) | 0 | 0 | 0 | 0 | 0 | 0 | 1 | 0 |
| Chudner, 2019b (16) | 0 | 0 | 0 | 1 | 0 | 1 | 0 | 0 |
| Ciecko, 2023 (17) | 0 | 0 | 0 | 1 | 0 | 0 | 0 | 0 |
| Devillers, 2023 (19) | 0 | 0 | 0 | 0 | 0 | 0 | 1 | 0 |
| Donaghy, 2019 (22) | 0 | 0 | 0 | 1 | 1 | 1 | 0 | 0 |
| Donaghy, 2023 (23) | 0 | 0 | 0 | 0 | 0 | 1 | 0 | 0 |
| Duncan, 2021 (24) | 0 | 1 | 0 | 0 | 1 | 0 | 0 | 0 |
| Esber, 2023 (25) | 0 | 0 | 0 | 1 | 0 | 0 | 0 | 0 |
| Garrett, 2022 (26) | 1 | 1 | 0 | 0 | 0 | 0 | 0 | 0 |
| Greenhalgh, 2022 (27) | 0 | 0 | 0 | 0 | 0 | 1 | 0 | 0 |
| Javanparast, 2021a (30) | 0 | 0 | 1 | 0 | 1 | 0 | 0 | 0 |
| Kludacz-Alessandri, 2021 (32) | 0 | 0 | 0 | 0 | 0 | 0 | 1 | 0 |
| Kowalski, 2018 (33) | 0 | 0 | 0 | 0 | 1 | 1 | 0 | 0 |
| Leng, 2016 (34) | 0 | 0 | 1 | 1 | 0 | 1 | 0 | 1 |
| Mathew, 2021 (37) | 0 | 0 | 0 | 0 | 0 | 0 | 1 | 0 |
| Mohan, 2022 (40) | 0 | 0 | 0 | 0 | 0 | 0 | 1 | 1 |
| Poitras, 2022 (42) | 0 | 0 | 0 | 0 | 1 | 1 | 0 | 0 |
| Powell, 2017 (43) | 0 | 0 | 0 | 0 | 0 | 0 | 1 | 1 |
| Reed, 2020 (44) | 0 | 0 | 1 | 0 | 0 | 0 | 0 | 0 |
| von Weinrich, 2022 (46) | 0 | 0 | 0 | 0 | 0 | 1 | 0 | 0 |

Note: the cell takes the value 1 if the article deals with the teleconsultation determinant of the column.

Additional file 12 – Results - Convenience for the patient

| Author first name, year | Convenience for the patient | | | | | | | | | |
| --- | --- | --- | --- | --- | --- | --- | --- | --- | --- | --- |
|  | Time-related | | | | | | Choice of location for teleconsultation | Emotional component (e.g., stress, anxiety) | Inclement weather | Others (Convenience for the patients) |
|  | Teleconsultation overcomes distances | Flexibility of schedule | Waiting time until the next available consultation appointment | Waiting time on appointment day | Delay in care | General/unspecified time saving |  |  |  |  |
| Aghajafari, 2022 (3) | 1 | 0 | 0 | 1 | 0 | 0 | 0 | 0 | 0 | 0 |
| Anderson, 2021 (4) | 1 | 0 | 0 | 0 | 0 | 0 | 1 | 0 | 0 | 0 |
| Atherton, 2018 (5) | 0 | 0 | 0 | 0 | 0 | 1 | 0 | 0 | 0 | 1 |
| Bali, 2007 (6) | 1 | 0 | 0 | 1 | 0 | 0 | 0 | 1 | 0 | 1 |
| Ball, 2018 (7) | 1 | 0 | 1 | 1 | 0 | 0 | 0 | 0 | 0 | 0 |
| Bhatia, 2022 (8) | 1 | 0 | 1 | 1 | 0 | 1 | 0 | 1 | 1 | 0 |
| Binder-Olibrowska, 2022 (9) | 1 | 0 | 1 | 1 | 0 | 0 | 0 | 0 | 0 | 0 |
| Bittleston, 2022 (10) | 1 | 1 | 1 | 0 | 0 | 1 | 1 | 1 | 0 | 0 |
| Buchanan, 2021 (12) | 0 | 0 | 1 | 0 | 0 | 0 | 0 | 0 | 0 | 0 |
| Burton, 2022 (13) | 1 | 0 | 0 | 1 | 0 | 0 | 0 | 1 | 0 | 0 |
| Chudner, 2019a (15) | 1 | 0 | 1 | 1 | 0 | 0 | 0 | 0 | 1 | 0 |
| Chudner, 2019b (16) | 0 | 0 | 1 | 1 | 0 | 0 | 0 | 0 | 0 | 0 |
| Curtis, 2021 (18) | 1 | 1 | 0 | 1 | 0 | 0 | 0 | 0 | 0 | 0 |
| Dixon, 2009 (21) | 0 | 0 | 0 | 0 | 0 | 0 | 0 | 1 | 0 | 0 |
| Donaghy, 2019 (22) | 1 | 1 | 1 | 1 | 0 | 0 | 0 | 0 | 0 | 0 |
| Garrett, 2022 (26) | 0 | 1 | 0 | 1 | 0 | 0 | 1 | 0 | 0 | 0 |
| Greenhalgh, 2022 (27) | 0 | 0 | 0 | 0 | 0 | 0 | 1 | 0 | 0 | 0 |
| Han, 2022 (28) | 0 | 0 | 1 | 1 | 0 | 0 | 0 | 1 | 0 | 0 |
| Javanparast, 2021a (30) | 1 | 0 | 1 | 0 | 0 | 0 | 0 | 0 | 0 | 0 |
| Kludacz-Alessandri, 2021 (32) | 0 | 0 | 1 | 0 | 0 | 0 | 0 | 0 | 0 | 0 |
| Leng, 2016 (34) | 1 | 1 | 1 | 0 | 0 | 1 | 1 | 0 | 1 | 0 |
| Mangalji, 2022 (35) | 0 | 0 | 0 | 1 | 0 | 0 | 0 | 0 | 0 | 0 |
| Manski-Nankervis, 2022 (36) | 1 | 0 | 0 | 0 | 0 | 1 | 0 | 0 | 0 | 0 |
| McKinstry, 2009 (38) | 1 | 0 | 0 | 0 | 0 | 1 | 0 | 1 | 0 | 0 |
| Mohan, 2022 (40) | 0 | 0 | 0 | 0 | 0 | 1 | 0 | 0 | 0 | 0 |
| Payne, 2001 (41) | 0 | 0 | 1 | 0 | 0 | 1 | 0 | 0 | 0 | 0 |
| Poitras, 2022 (42) | 1 | 0 | 0 | 0 | 1 | 1 | 0 | 0 | 0 | 0 |
| Powell, 2017 (43) | 1 | 1 | 0 | 1 | 0 | 0 | 0 | 1 | 0 | 0 |
| Reed, 2020 (44) | 1 | 0 | 0 | 0 | 0 | 0 | 0 | 0 | 0 | 0 |
| Rose, 2021 (45) | 0 | 0 | 0 | 0 | 0 | 1 | 1 | 0 | 0 | 0 |
| von Weinrich, 2022 (46) | 0 | 0 | 1 | 1 | 0 | 0 | 0 | 0 | 0 | 0 |

Note: the cell takes the value 1 if the article deals with the teleconsultation determinant of the column.

Additional file 13 – Results - Patient-centered care

| Author first name, year | Patient-centered care | | | | | | | | | | | | |
| --- | --- | --- | --- | --- | --- | --- | --- | --- | --- | --- | --- | --- | --- |
|  | Communication between patient and provider | Intrinsic value of face-to-face appointment | Continuity of healthcare | Relationship between patient and provider | Provider involvement in consultation | | | Patient involvement in consultation | Patient's understanding of his/her condition | Access to health information | Management of patient's health issues/concerns | Disagreement between patient and provider | Others (Patient-centered care) |
|  |  |  |  |  | Explanation provided by practitioners | Provider's attention/focus | Provider's general involvement |  |  |  |  |  |  |
| Abraham, 2022 (1) | 0 | 0 | 0 | 0 | 0 | 0 | 0 | 0 | 0 | 0 | 1 | 0 | 0 |
| Adams, 2023 (2) | 0 | 0 | 1 | 1 | 0 | 0 | 0 | 0 | 0 | 0 | 0 | 1 | 0 |
| Aghajafari, 2022 (3) | 0 | 0 | 0 | 1 | 0 | 0 | 0 | 0 | 0 | 0 | 0 | 0 | 0 |
| Assing Hvidt, 2022 (29) | 0 | 0 | 0 | 1 | 0 | 1 | 0 | 1 | 0 | 0 | 0 | 0 | 1 |
| Atherton, 2018 (5) | 0 | 0 | 0 | 1 | 0 | 0 | 0 | 0 | 0 | 0 | 0 | 0 | 0 |
| Bali, 2007 (6) | 0 | 0 | 1 | 0 | 0 | 0 | 0 | 0 | 1 | 0 | 0 | 0 | 0 |
| Ball, 2018 (7) | 1 | 1 | 1 | 1 | 0 | 0 | 0 | 0 | 0 | 1 | 0 | 0 | 0 |
| Bhatia, 2022 (8) | 0 | 1 | 0 | 1 | 0 | 1 | 0 | 0 | 0 | 0 | 0 | 0 | 1 |
| Binder-Olibrowska, 2022 (9) | 0 | 0 | 0 | 1 | 0 | 0 | 1 | 0 | 0 | 0 | 0 | 0 | 1 |
| Bittleston, 2022 (10) | 0 | 0 | 0 | 1 | 0 | 1 | 0 | 0 | 0 | 0 | 1 | 0 | 1 |
| Brown, 1995 (11) | 0 | 0 | 0 | 0 | 0 | 0 | 1 | 0 | 0 | 0 | 0 | 0 | 0 |
| Burton, 2022 (13) | 0 | 0 | 0 | 0 | 0 | 0 | 0 | 0 | 0 | 0 | 0 | 0 | 1 |
| Chudner, 2019a (15) | 0 | 0 | 0 | 1 | 0 | 1 | 0 | 0 | 0 | 0 | 0 | 0 | 0 |
| Chudner, 2019b (16) | 0 | 0 | 0 | 1 | 0 | 1 | 0 | 0 | 0 | 0 | 0 | 0 | 0 |
| Ciecko, 2023 (17) | 0 | 0 | 0 | 0 | 1 | 0 | 0 | 0 | 0 | 0 | 1 | 0 | 0 |
| Curtis, 2021 (18) | 1 | 1 | 0 | 1 | 1 | 0 | 0 | 0 | 0 | 0 | 0 | 0 | 0 |
| Dixon, 2008 (20) | 0 | 0 | 0 | 0 | 0 | 0 | 1 | 0 | 0 | 0 | 0 | 0 | 0 |
| Donaghy, 2019 (22) | 0 | 0 | 0 | 1 | 0 | 0 | 0 | 0 | 0 | 0 | 0 | 0 | 0 |
| Donaghy, 2023 (23) | 0 | 0 | 1 | 1 | 0 | 0 | 1 | 0 | 0 | 0 | 0 | 0 | 0 |
| Duncan, 2021 (24) | 0 | 0 | 1 | 0 | 0 | 0 | 0 | 0 | 0 | 0 | 0 | 0 | 0 |
| Esber, 2023 (25) | 0 | 0 | 0 | 1 | 0 | 0 | 0 | 0 | 0 | 0 | 0 | 0 | 0 |
| Garrett, 2022 (26) | 0 | 0 | 0 | 1 | 0 | 0 | 0 | 0 | 0 | 0 | 0 | 0 | 0 |
| Greenhalgh, 2022 (27) | 0 | 0 | 0 | 1 | 0 | 0 | 0 | 0 | 0 | 0 | 0 | 0 | 1 |
| Han, 2022 (28) | 0 | 0 | 0 | 0 | 0 | 1 | 0 | 0 | 0 | 0 | 0 | 0 | 0 |
| Javanparast, 2021a (30) | 0 | 0 | 1 | 1 | 0 | 0 | 0 | 0 | 0 | 0 | 1 | 0 | 0 |
| McKinstry, 2009 (38) | 0 | 0 | 1 | 1 | 0 | 0 | 0 | 0 | 0 | 0 | 1 | 0 | 0 |
| McKinstry, 2010 (39) | 0 | 0 | 0 | 1 | 0 | 0 | 1 | 0 | 0 | 0 | 1 | 0 | 0 |
| Payne, 2001 (41) | 0 | 0 | 0 | 0 | 0 | 1 | 0 | 0 | 0 | 0 | 0 | 0 | 0 |
| Poitras, 2022 (42) | 0 | 0 | 1 | 0 | 0 | 0 | 0 | 0 | 0 | 0 | 1 | 0 | 0 |
| Reed, 2020 (44) | 0 | 0 | 0 | 1 | 0 | 0 | 0 | 0 | 0 | 0 | 0 | 0 | 0 |
| Rose, 2021 (45) | 0 | 0 | 0 | 1 | 0 | 0 | 1 | 0 | 0 | 0 | 1 | 0 | 0 |
| von Weinrich, 2022 (46) | 0 | 0 | 0 | 1 | 0 | 0 | 0 | 0 | 0 | 0 | 0 | 0 | 0 |

Note: the cell takes the value 1 if the article deals with the teleconsultation determinant of the column.

Additional file 14 – Results - Institution-related

| Author first name, year | Institution-related | | | | | | | | | | | | |
| --- | --- | --- | --- | --- | --- | --- | --- | --- | --- | --- | --- | --- | --- |
|  | Role and value of general practice | Legal context | | Economic and financial aspects | | Patient's awareness about teleconsultation | Access to care | | | | Equity and fairness | | |
|  |  | Availability of teleconsultation / Modes of teleconsultation available | Legislation and guidelines (protocol/guidance) | Reimbursement by health insurance | Efficiency/resources allocation/saving |  | Financial barriers | | Ease of Ease of prescription retrieval and access to medication retrieval | Lessened barriers to access to care / general access to care | Digital exclusion | Income-based exclusion | General equity of care delivery |
|  |  |  |  |  |  |  | Cost of TC/F2F | Travel Cost |  |  |  |  |  |
| Adams, 2023 (2) | 0 | 0 | 0 | 0 | 0 | 1 | 0 | 0 | 0 | 0 | 0 | 0 | 0 |
| Assing Hvidt, 2022 (29) | 1 | 0 | 0 | 0 | 0 | 0 | 0 | 0 | 0 | 0 | 1 | 0 | 0 |
| Atherton, 2018 (5) | 0 | 0 | 0 | 0 | 0 | 1 | 0 | 0 | 0 | 0 | 0 | 0 | 0 |
| Bali, 2007 (6) | 0 | 0 | 0 | 0 | 0 | 0 | 1 | 0 | 0 | 1 | 0 | 1 | 0 |
| Ball, 2018 (7) | 0 | 1 | 0 | 0 | 1 | 1 | 0 | 0 | 1 | 1 | 0 | 0 | 1 |
| Bhatia, 2022 (8) | 0 | 0 | 1 | 0 | 1 | 0 | 0 | 0 | 0 | 0 | 0 | 0 | 0 |
| Binder-Olibrowska, 2022 (9) | 0 | 0 | 0 | 0 | 0 | 0 | 0 | 0 | 1 | 0 | 0 | 0 | 0 |
| Bittleston, 2022 (10) | 0 | 0 | 0 | 0 | 0 | 0 | 1 | 0 | 0 | 0 | 0 | 0 | 0 |
| Brown, 1995 (11) | 0 | 0 | 0 | 0 | 0 | 1 | 0 | 0 | 0 | 0 | 0 | 0 | 0 |
| Buchanan, 2021 (12) | 0 | 0 | 0 | 0 | 0 | 0 | 1 | 0 | 1 | 0 | 0 | 0 | 0 |
| Burton, 2022 (13) | 0 | 0 | 0 | 0 | 0 | 0 | 0 | 0 | 0 | 0 | 0 | 1 | 0 |
| Chudner, 2019a (15) | 0 | 0 | 0 | 0 | 0 | 0 | 0 | 1 | 0 | 0 | 0 | 0 | 0 |
| Garrett, 2022 (26) | 0 | 0 | 0 | 0 | 0 | 0 | 1 | 0 | 0 | 0 | 0 | 0 | 0 |
| Greenhalgh, 2022 (27) | 0 | 0 | 0 | 0 | 0 | 1 | 0 | 0 | 0 | 0 | 0 | 0 | 0 |
| Han, 2022 (28) | 0 | 0 | 0 | 0 | 0 | 0 | 0 | 0 | 0 | 1 | 0 | 0 | 0 |
| Javanparast, 2021a (30) | 0 | 1 | 0 | 0 | 0 | 0 | 1 | 0 | 0 | 0 | 0 | 0 | 0 |
| Leng, 2016 (34) | 0 | 0 | 0 | 0 | 0 | 0 | 1 | 1 | 0 | 0 | 0 | 0 | 0 |
| McKinstry, 2009 (38) | 0 | 0 | 0 | 0 | 0 | 0 | 0 | 0 | 1 | 0 | 0 | 0 | 0 |
| Mohan, 2022 (40) | 0 | 0 | 0 | 0 | 0 | 0 | 1 | 0 | 0 | 0 | 0 | 0 | 0 |
| Payne, 2001 (41) | 0 | 0 | 0 | 0 | 0 | 0 | 0 | 0 | 0 | 1 | 0 | 0 | 0 |
| Poitras, 2022 (42) | 0 | 0 | 0 | 0 | 0 | 0 | 1 | 0 | 0 | 0 | 0 | 0 | 0 |
| Powell, 2017 (43) | 0 | 0 | 0 | 0 | 0 | 0 | 1 | 0 | 0 | 0 | 0 | 0 | 0 |
| Reed, 2020 (44) | 0 | 0 | 0 | 0 | 0 | 0 | 1 | 0 | 0 | 0 | 0 | 0 | 0 |
| Rose, 2021 (45) | 0 | 0 | 0 | 1 | 0 | 1 | 1 | 0 | 1 | 1 | 0 | 0 | 0 |

Note: the cell takes the value 1 if the article deals with the teleconsultation determinant of the column.

Additional file 15 – Number of occurrences by theme or subtheme

| Themes / Sub-themes | | Occurrences |
| --- | --- | --- |
| Consultation purpose | | 94 |
| Patient-centered care | Relationship | 22 |
| Patient's demographic profile | Age | 18 |
| Time-related | Teleconsultation overcomes distances | 18 |
|  | Waiting time on appointment day | 15 |
| Patient-centered care | Provider involvement in consultation | 15 |
| Duration of consultation | | 14 |
| Time-related | Waiting time until the next available consultation appointment | 14 |
| Cost of TC/F2F | | 11 |
| Complementarity/Substitutability between modalities of consultation |  | 11 |
| Teleconsultation undermines the accuracy/reliability | Lack of/difficulties in physical examinations | 10 |
| Patient's demographic profile | Family/Having children | 10 |
| Patient's attitude towards teleconsultation | Patient's prior preferences for face-to-face consultation over teleconsultation | 10 |
| Time-related | General/unspecified time saving | 10 |
| TC technology related | Technical issues | 10 |
| Teleconsultation undermines the accuracy/reliability | Risk of misunderstanding/misdiagnosis/error | 9 |
| TC technology related | Privacy, confidentiality, and security issues | 9 |
| Patient's attitude towards teleconsultation | Patient's experience with teleconsultation | 9 |
|  | Patient's prior preferences for video-consultation over phone-consultation | 9 |
| Emotional component (e.g., stress, anxiety) | | 9 |
| Teleconsultation undermines the accuracy/reliability | Lack of verbal, non-verbal, and behavioral cues | 8 |
| Patient-centered care | Management of patient's health issues/concerns | 8 |
|  | Continuity of healthcare (better management of patient's healthcare pathway) | 8 |
| Consultation setting | Ease of appointment booking | 7 |
| Patient's socioeconomic profile | Occupation status | 7 |
| Patient's demographic profile | Gender (female) | 7 |
| General patient's abilities and knowledge | | 7 |
| Consultation setting | Involvement of a third party | 6 |
| TC technology related | Equipment required for teleconsultation | 6 |
| Patient's medical condition | Hearing problems | 6 |
|  | Mental health/cognitive problems | 6 |
| Patient's ability to use technology | | 6 |
| Choice of location for teleconsultation | | 6 |
| Time-related | Flexibility of schedule | 6 |
| Consultation setting | Virtual waiting room | 5 |
| Patient's socioeconomic profile | Living remotely | 5 |
| Patient's demographic profile | Language | 5 |
| Patient's ability to communicate with technology | | 5 |
| Lessened barriers to access to care / general access to care | | 5 |
| Patient's medical condition | Restricted mobility | 4 |
| Ease of prescription retrieval | | 4 |
| Patient's medical condition | Disabled | 4 |
| General provider's abilities and knowledge | | 3 |
| Provider's ability to communicate | | 3 |
| Patient's socioeconomic profile | Education level | 3 |
| Patient's demographic profile | Ethnicity | 3 |
| Patient's medical condition | Visual problems | 3 |
|  | Chronic condition | 3 |
| Patient's ability to communicate | | 3 |
| Patient's attitude towards teleconsultation | General attitudes towards teleconsultation | 3 |
| Inclement weather | | 3 |
| Teleconsultation undermines the accuracy/reliability | Lack of intimacy | 3 |
| Consultation setting | Triaging | 2 |
| Patient's socioeconomic profile | Income | 2 |
| Travel Cost | | 2 |
| Income-based exclusion | | 2 |
| Consultation setting | Possibility to send documents (e.g., test results, photos, videos) | 1 |
| Patient's socioeconomic profile | Being uninsured | 1 |
| Patient's medical condition | Regular medication | 1 |
| Time-related | Delay in diagnosis and treatment | 1 |
| Ease of access to medication | | 1 |
| Digital exclusion | | 1 |

Additional file 16 – Number of occurrences - Consultation purposes only

| Themes / Sub-themes | | Occurrences |
| --- | --- | --- |
| Health issues | Complexity and severity of the health issue | 11 |
|  | Mental health issues | 10 |
|  | Follow-up/Routine consultation | 9 |
| Administrative and documentation | Prescription renewal/refill | 9 |
| Assessment and guidance | Request tests and discussing test results | 9 |
| Health issues | Sensitive or personal issues | 7 |
| Assessment and guidance | Need a visual/physical examination | 6 |
| Health issues | Dermatological conditions (e.g., rashes) | 5 |
| Assessment and guidance | Discussing treatment | 4 |
| Health issues | Emergency health issues | 3 |
| Assessment and guidance | General advice | 3 |
| Health issues | New health issues | 2 |
|  | Child health issues | 2 |
| Administrative and documentation | Obtaining certificate (e.g., sick leave) | 2 |
|  | Referring (e.g., specialist, lab) | 1 |
| Assessment and guidance | Diagnosis | 2 |
|  | Others (Assessment and guidance) | 1 |
| Health issues | Mobility issues | 1 |


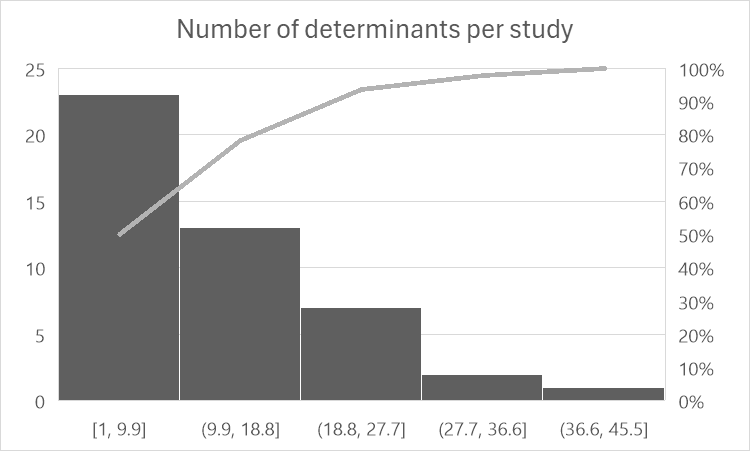
Additional file 17 – Number of determinants of teleconsultation use per study

| Author first name, year | Number of determinants |
| --- | --- |
| Abraham, 2022 (1) | 2 |
| Adams, 2023 (2) | 19 |
| Aghajafari, 2022 (3) | 11 |
| Anderson, 2021 (4) | 9 |
| Atherton, 2018 (5) | 10 |
| Bali, 2007 (6) | 24 |
| Ball, 2018 (7) | 32 |
| Bhatia, 2022 (8) | 34 |
| Binder-Olibrowska, 2022 (9) | 25 |
| Bittleston, 2022 (10) | 17 |
| Brown, 1995 (11) | 7 |
| Buchanan, 2021 (12) | 6 |
| Burton, 2022 (13) | 10 |
| Chen, 2022 (14) | 7 |
| Chudner, 2019a (15) | 7 |
| Chudner, 2019b (16) | 7 |
| Ciecko, 2023 (17) | 5 |
| Curtis, 2021 (18) | 18 |
| Devillers, 2023 (19) | 4 |
| Dixon, 2008 (20) | 2 |
| Dixon, 2009 (21) | 2 |
| Donaghy, 2019 (22) | 18 |
| Donaghy, 2023 (23) | 7 |
| Duncan, 2021 (24) | 8 |
| Esber, 2023 (25) | 7 |
| Garrett, 2022 (26) | 23 |
| Greenhalgh, 2022 (27) | 10 |
| Han, 2022 (28) | 17 |
| Assing Hvidt, 2022 (29) | 22 |
| Javanparast, 2021a (30) | 18 |
| Javanparast, 2021b (31) | 4 |
| Kludacz-Alessandri, 2021 (32) | 5 |
| Kowalski, 2018 (33) | 3 |
| Leng, 2016 (34) | 38 |
| Mangalji, 2022 (35) | 1 |
| Manski-Nankervis, 2022 (36) | 5 |
| Mathew, 2021 (37) | 2 |
| McKinstry, 2009 (38) | 23 |
| McKinstry, 2010 (39) | 6 |
| Mohan, 2022 (40) | 10 |
| Payne, 2001 (41) | 9 |
| Poitras, 2022 (42) | 20 |
| Powell, 2017 (43) | 17 |
| Reed, 2020 (44) | 10 |
| Rose, 2021 (45) | 17 |
| von Weinrich, 2022 (46) | 5 |

References

1. Abraham HN, Acuff C, Brauer B, Nabaty R, Opara IN, Levine DL. Patient Satisfaction With Medical and Social Concerns Addressed During Telemedicine Visits. Cureus. 2022;14(12):e32529.

2. Adams AM, Williams KKA, Langill JC, Arsenault M, Leblanc I, Munro K, et al. Telemedicine perceptions and experiences of socially vulnerable households during the early stages of the COVID-19 pandemic: a qualitative study. CMAJ Open. 2023;11(2):E219–26.

3. Aghajafari F, Santana MJ, Abboud R, Claussen C. Impact of COVID-19 on primary care: Addressing health concerns and older patient experience of virtual care. Ann Fam Med. 2023;21:1–2.

4. Anderson J, Walsh J, Anderson M, Burnley R. Patient Satisfaction With Remote Consultations in a Primary Care Setting. Cureus. 2021;13(9):e17814.

5. Atherton H, Brant H, Ziebland S, Bikker A, Campbell J, Gibson A, et al. Alternatives to the face-to-face consultation in general practice: focused ethnographic case study. Br J Gen Pr. 2018;68(669):e293–300.

6. Bali S, Singh AJ. Mobile phone consultation for community health care in rural north India. J Telemed Telecare. 2007;13(8):421–4.

7. Ball SL, Newbould J, Corbett J, Exley J, Pitchforth E, Roland M. Qualitative study of patient views on a “telephone-first” approach in general practice in England: speaking to the GP by telephone before making face-to-face appointments. BMJ Open. 2018;8(12):e026197.

8. Bhatia R, Gilliam E, Aliberti G, Pinheiro A, Karamourtopoulos M, Davis RB, et al. Older adults’ perspectives on primary care telemedicine during the COVID‐19 pandemic. J Am Geriatr Soc. 2022;70(12):3480–92.

9. Binder-Olibrowska KW, Wrzesińska MA, Godycki-Ćwirko M. Is Telemedicine in Primary Care a Good Option for Polish Patients with Visual Impairments Outside of a Pandemic? Int J Env Res Public Health. 2022;19(11).

10. Bittleston H, Goller JL, Temple-Smith M, Hocking JS, Coombe J. Telehealth for sexual and reproductive health issues: a qualitative study of experiences of accessing care during COVID-19. Sex Health. 2022;19(5):473–8.

11. Brown A, Armstrong D. Telephone consultations in general practice: an additional or alternative service? Br J Gen Pr. 1995;45(401):673–5.

12. Buchanan J, Roope LSJ, Morrell L, Pouwels KB, Robotham JV, Abel L, et al. Preferences for Medical Consultations from Online Providers: Evidence from a Discrete Choice Experiment in the United Kingdom. Appl Health Econ Health Policy. 2021;19(4):521–35.

13. Burton L, Rush KL, Smith MA, Davis S, Rodriguez Echeverria P, Suazo Hidalgo L, et al. Empowering Patients Through Virtual Care Delivery: Qualitative Study With Micropractice Clinic Patients and Health Care Providers. JMIR Form Res. 2022;6(4):e32528.

14. Chen K, Zhang C, Gurley A, Akkem S, Jackson H. Primary care utilization among telehealth users and non-users at a large urban public healthcare system. PLoS ONE. 2022;17(8):1–6.

15. Chudner I, Goldfracht M, Goldblatt H, Drach-Zahavy A, Karkabi K. Video or In-Clinic Consultation? Selection of Attributes as Preparation for a Discrete Choice Experiment Among Key Stakeholders. The Patient. 2019 Feb;12(1):69–82.

16. Chudner I, Drach-Zahavy A, Karkabi K. Choosing Video Instead of In-Clinic Consultations in Primary Care in Israel: Discrete Choice Experiment Among Key Stakeholders-Patients, Primary Care Physicians, and Policy Makers. Value Health J Int Soc Pharmacoeconomics Outcomes Res. 2019 Oct;22(10):1187–96.

17. Ciećko W, Labunets K, Wojnarowska M, Bosek D, Skwierawska J, Bandurski T, et al. How COVID-19 Broke the Barriers Related to the Implementation of Telecare-Patients’ Experiences with a New form of Providing Health Services in Primary Health Care. Healthc Basel Switz [Internet]. 2023;11(4). Available from: https://search.ebscohost.com/login.aspx?direct=true&db=mnh&AN=36833033&site=ehost-live

18. Curtis M, Duncan R, Meng Jing, Kim A, Lu VT, Redshaw J, et al. “Not a perfect situation, but...” A single-practice survey of patient experience of phone consultations during COVID-19 Alert Level 4 in New Zealand. N Z Med J. 2021;134(1544):35–48.

19. Devillers N, Trombert B, Frappe P, Pernoud L, Laval B. Teleconsultation in general practice. Overview and patients’ satisfaction. Exerc- Rev Francoph Med Gen. 2023;(190):52–7.

20. Dixon RF, Stahl JE. Virtual visits in a general medicine practice: a pilot study. Telemed J E Health. 2008;14(6):525–30.

21. Dixon RF, Stahl JE. A randomized trial of virtual visits in a general medicine practice. J Telemed Telecare. 2009;15(3):115–7.

22. Donaghy E, Atherton H, Hammersley V, McNeilly H, Bikker A, Robbins L, et al. Acceptability, benefits, and challenges of video consulting: a qualitative study in primary care. Br J Gen Pr. 2019 Sep;69(686):e586–94.

23. Donaghy E, Still F, Frost H, Lutte J, Shenkin SD, Jones HE, et al. GP-led adapted comprehensive geriatric assessment for frail older people: a multi-methods evaluation of the “Living Well Assessment” quality improvement project in Scotland. BJGP Open [Internet]. 2023;7(1). Available from: https://search.ebscohost.com/login.aspx?direct=true&db=mnh&AN=36564081&site=ehost-live

24. Duncan LJ, Cheng KFD. Public perception of NHS general practice during the first six months of the COVID-19 pandemic in England. F1000Research. 2021;10:279.

25. Esber A, Teufel M, Jahre L, In der Schmitten J, Skoda EM, Bäuerle A. Predictors of patients’ acceptance of video consultation in general practice during the coronavirus disease 2019 pandemic applying the unified theory of acceptance and use of technology model. Digit Health. 2023;9:20552076221149317.

26. Garrett SM, Rose SB, McKinlay EM. Young people talk about primary care and telehealth: A survey of 15‐ to 25‐year olds in the Wellington region of New Zealand. Health Soc Care Community. 2022;30(6):e6345–55.

27. Greenhalgh T, Ladds E, Hughes G, Moore L, Wherton J, Shaw SE, et al. Why do GPs rarely do video consultations? qualitative study in UK general practice. Br J Gen Pract J R Coll Gen Pract. 2022;72(718):e351–60.

28. Dongqi H, Heshmat Y, Geiskkovitch DY, Tan Z, Neustaedter C. A Scenario-Based Study of Doctors and Patients on Video Conferencing Appointments from Home. ACM Trans Comput-Hum Interact TOCHI. 2022;29(5):1–35.

29. Assing Hvidt E, Christensen NP, Grønning A, Jepsen C, Lüchau EC. What are patients’ first-time experiences with video consulting? A qualitative interview study in Danish general practice in times of COVID-19. BMJ Open. 2022;12(4):e054415.

30. Javanparast S, Roeger L, Kwok Y, Reed RL. The experience of Australian general practice patients at high risk of poor health outcomes with telehealth during the COVID-19 pandemic: a qualitative study. BMC Fam Pract. 2021;22(1):69.

31. Javanparast S, Roeger L, Reed RL. Experiences of patients with chronic diseases of access to multidisciplinary care during COVID-19 in South Australia. Aust Health Rev. 2021;45(5):525–32.

32. Kludacz-Alessandri M, Hawrysz L, Korneta P, Gierszewska G, Pomaranik W, Walczak R. The impact of medical teleconsultations on general practitioner-patient communication during COVID-19: A case study from Poland. Vol. 16, PLOS ONE. 1160 BATTERY STREET, STE 100, SAN FRANCISCO, CA 94111 USA: PUBLIC LIBRARY SCIENCE; 2021.

33. Kowalski A, Yoshioka K, Mancuso A, Moore R. Factors and Preferences in Patient Selection and Location of Care. Health CARE Manag. 2018;37(4):311–6.

34. Leng S, MacDougall M, McKinstry B. The acceptability to patients of video-consulting in general practice: semi-structured interviews in three diverse general practices. J Innov Health Inf. 2016;23(2):141.

35. Mangalji A, Chahal P, Cherukupalli A. Evaluating patient perceptions of quality of care through telemedicine during the COVID-19 pandemic. Br Columbia Med J. 2022;64(6):265–7.

36. Manski-Nankervis JA, Davidson S, Hiscock H, Hallinan C, Ride J, Lingam V, et al. Primary care consumers’ experiences and opinions of a telehealth consultation delivered via video during the COVID-19 pandemic. Aust J Prim Health. 2022;28(3):224–31.

37. Mathew T, Lee PC, Ianno DJ, Benson J. Telehealth and Australian general practice in 2020: A survey exploring patients’ perspectives in the Adelaide Hills. Aust J Gen Pract. 2021;50(10):754–9.

38. McKinstry B, Watson P, Pinnock H, Heaney D, Sheikh A. Telephone consulting in primary care: a triangulated qualitative study of patients and providers. Br J Gen Pr. 2009;59(563):e209-18.

39. McKinstry B, Hammersley V, Burton C, Pinnock H, Elton R, Dowell J, et al. The quality, safety and content of telephone and face-to-face consultations: a comparative study. Qual Saf Health Care. 2010;19(4):298–303.

40. Mohan S, Lin W, Orozco FR, Robinson J, Mahoney A. Patient Perceptions of Video Visits in a Fee-for-Service Model. J Am Board Fam Med JABFM. 2022;35(3):497–506.

41. Payne F, Shipman C, Dale J. Patients’ experiences of receiving telephone advice from a GP co-operative. Fam Pr. 2001;18(2):156–60.

42. Poitras ME, Poirier MD, Couturier Y, T Vaillancourt V, Cormier C, Gauthier G, et al. Chronic conditions patient’s perception of post-COVID-19 pandemic teleconsulting continuation in primary care clinics: a qualitative descriptive study. BMJ Open. 2022;12(12):e066871.

43. Powell RE, Henstenburg JM, Cooper G, Hollander JE, Rising KL. Patient Perceptions of Telehealth Primary Care Video Visits. Vol. 15, ANNALS OF FAMILY MEDICINE. 11400 TOMAHAWK CREEK PARKWAY, LEAWOOD, KS 66211-2672 USA: ANNALS FAMILY MEDICINE; 2017. p. 225–9.

44. Reed ME, Huang J, Graetz I, Lee C, Muelly E, Kennedy C, et al. Patient Characteristics Associated With Choosing a Telemedicine Visit vs Office Visit With the Same Primary Care Clinicians. JAMA Netw Open. 2020;3(6):e205873–e205873.

45. Rose S, Hurwitz HM, Mercer MB, Hizlan S, Gali K, Yu PC, et al. Patient Experience in Virtual Visits Hinges on Technology and the Patient-Clinician Relationship: A Large Survey Study With Open-ended Questions. J Med Internet Res. 2021;23(6):e18488.

46. von Weinrich P, Kong Q, Liu Y. Would you zoom with your doctor? A discrete choice experiment to identify patient preferences for video and in-clinic consultations in German primary care. J Telemed Telecare. 2022 Aug 2;1357633X221111975.
